# Comparative physicochemical characterization and sensory profiling of Western Algerian and Polish honeys

**DOI:** 10.1371/journal.pone.0334514

**Published:** 2025-10-17

**Authors:** Dalila Bereksi-Reguig, Hocine Allali, Salim Bouchentouf, Nessrine Kazi Tani, Grażyna Kowalska, Dariusz Kowalczyk, Jakub Wyrostek, Ewelina Zielińska, Radosław Kowalski

**Affiliations:** 1 Department of Chemistry, Faculty of Sciences, Abou Bekr Belkaïd University, Tlemcen, Algeria; 2 Laboratory of Natural and Bioactive Substances (LASNABIO), Department of Chemistry, Faculty of Sciences, Abou Bekr Belkaïd University, Tlemcen, Algeria; 3 Doctor Tahar Moulay University of Saida, Saïda, Algeria; 4 Laboratory of Application of Electrolytes and Organic Poly-electrolytes (LCPO), Abou Bekr Belkaïd University, Tlemcen, Algeria; 5 Department of Tourism and Recreation, University of Life Sciences in Lublin, Lublin, Poland; 6 Department of Biochemistry and Food Chemistry, University of Life Sciences in Lublin, Lublin, Poland; 7 Department of Analysis and Evaluation of Food Quality, University of Life Sciences in Lublin, Lublin, Poland; State University of Bangladesh, BANGLADESH

## Abstract

**Background:**

An in-depth analysis was conducted on 37 honey samples from western Algeria representing diverse floral sources—lavender, rosemary, sweet white mustard, thyme, milk thistle, carob, orange, euphorbia, eucalyptus, camphor, jujube, sage, harmal, and multifloral blends. The objective was to evaluate their physicochemical properties and sensory characteristics, with Polish honeys serving as references.

**Methods:**

Key physicochemical traits were measured, including moisture, pH, free acidity, electrical conductivity, hydroxymethylfurfural (HMF), proline, specific optical rotation, sugar profile (fructose, glucose, sucrose), and colour in CIELAB space (L*, a*, b*, $C_{ab}^{*}$ and $h_{a}^{b^{\circ }}$). Sensory evaluation was performed using a five-point hedonic scale (+2 = “like very much” to –2 = “dislike very much”) and an 11-descriptor Check-All-That-Apply (CATA) questionnaire.

**Results:**

All values satisfied European quality limits (moisture 14.67–20.87%, pH 3.47–5.60, free acidity 8.00–40.33 meq/kg, conductivity 0.16–1.18 mS/cm, HMF 1.79–49.43 mg/kg, sucrose < 5 g/100 g, proline 265.95–1200.66 mg/kg). Polish samples scored higher for taste (+1.22 ± 0.42 vs + 0.18 ± 0.52; p = 0.009) and aroma (+0.72 ± 0.43 vs –0.26 ± 0.36; p = 0.016), whereas colour did not differ (p = 0.459). CATA indicated Algerian honeys were chiefly “mild” and “herbal”, contrasting with Polish “sweet” and “sharp” profiles. Principal-component analysis (PC1 + PC2 ≈ 65% variance) and hierarchical clustering defined three groups: (A) sweet aromatic (all Polish + four Algerian), (B) moderately mild, and (C) sharp–bitter–herbal. Selected Algerian varietals—rosemary (S29) and multifloral (S14)—matched Polish hedonic acceptance, highlighting their premium/exotic market potential.

**Conclusion:**

Western Algerian honeys exhibit high compositional quality and distinctive sensory signatures, supporting competitiveness in food and health applications.

## 1. Introduction

Honey is a natural product containing more than 200 substances, including sugars, proteins, amino acids, water, enzymes, organic acids, vitamins, minerals, phenolic compounds, and volatile constituents [1–3]. It is widely used as both a food and a natural sweetener without additives, with a long shelf life and suitability for culinary applications [4]. The composition of honey varies according to its botanical and geographical origins, which influence its physicochemical parameters such as colour, moisture, and acidity, and consequently its organoleptic properties [5,6].

According to the Codex Alimentarius, honey is defined as “the natural sweet substance produced by honey bees from the nectar of plants, from secretions of living parts of plants, or from excretions of plant-sucking insects” [7,8]. Beyond its nutritional and functional roles, honey has socioeconomic significance through beekeeping, which contributes to pollination, agricultural economies, rural development, and biodiversity conservation [9]. Bees and hive products are also increasingly recognised as bioindicators of environmental pollution [10].

Honeys are typically classified as monofloral or multifloral depending on whether they originate predominantly from one floral source or from several. Multifloral honeys, also known as “polyfloral” or “all-flower” honeys, result from the contribution of multiple plant species within a given region and season [11]. Increasing consumer demand for authenticated honeys has stimulated interest in characterising both monofloral and multifloral varieties [12,13].

The botanical origin of honey is primarily determined through melissopalynology, the analysis of pollen grains [14]. This is often complemented by physicochemical and sensory analyses to provide a more comprehensive characterisation [15]. Additionally, volatile compound profiling has emerged as a reliable and precise technique for verifying authenticity [16]. By integrating these methods, a robust and definitive framework is established for authenticating honey and its floral provenance. This multi-faceted approach ensures a complete and accurate determination of the product’s origin.

In Algeria, systematic data on beekeeping and honey production remain scarce. The available evidence suggests that the sector comprises approximately 1.2 million colonies and 20,000 beekeepers, with a substantial increase in honey production between 2002 and 2010. However, the mean yield per hive remains below 4 kg [9]. Western Algeria, with its Mediterranean–Saharan climate and diverse flora, offers conditions conducive to the production of honeys with distinctive physicochemical and sensory profiles.

Poland represents an established European honey market with well-documented physicochemical and sensory standards. Comparative analyses between Algerian and Polish honeys are therefore relevant for assessing quality and positioning Algerian varieties in international contexts. Recent studies have also shown that declared geographical origin influences consumer perception and willingness to pay more strongly than intrinsic taste attributes [17], underscoring the importance of linking physicochemical data with sensory evaluation.

This study aimed to characterise the physicochemical parameters (moisture, pH, free acidity, electrical conductivity, hydroxymethylfurfural content, proline, specific optical rotation, sugar composition, and colour) of 37 honey samples from western Algeria of diverse botanical origins, and to evaluate their sensory properties in comparison with four Polish reference honeys. The overarching goal was to assess the quality profiles of Algerian honeys and their potential positioning relative to established European references.

## 2. Materials and methods

### 2.1 Chemicals and reagents

The chemicals employed comprised hydroxymethylfurfural (HMF, ≥ 99%) and proline, both supplied by Sigma-Aldrich® (Chemie GmbH, Taufkirchen, Germany). Ninhydrin, ethylene glycol monomethyl ether, and 2-propanol were obtained from Sigma-Aldrich® (Darmstadt, Germany). All reagents were of analytical-grade purity.

### 2.2 Honey samples

**Fig 1:**
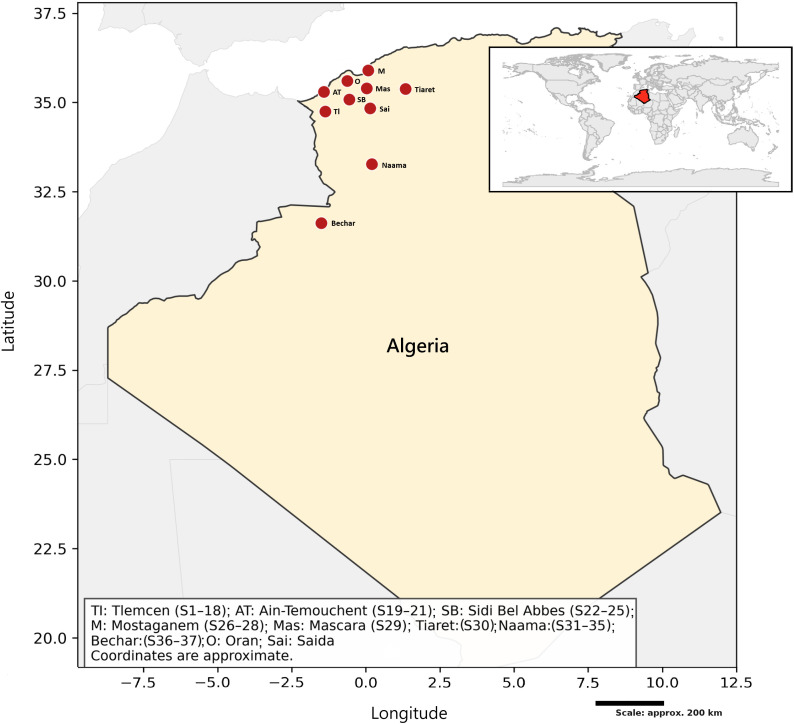
Map of western Algeria depicting the distribution of the studied honey samples.

Thirty-seven honey samples, identified by codes S1–S37, were collected from experienced beekeepers across different regions of western Algeria, including Tlemcen, Ain-Temouchent, Sidi Bel Abbes, Mostaganem, Mascara, Tiaret, Naâma, and Bechar (Fig 1). The botanical sources of these samples included lavender (*Lavandula vera* D.C.), rosemary (*Rosmarinus officinalis* L.), sweet white mustard (*Sinapis alba* L.), thyme (*Thymus vulgaris* L.), milk thistle (*Silybum marianum* (L.) Gaertn.), carob (*Ceratonia siliqua* L.), orange (*Citrus sinensis* L.), euphorbia (*Euphorbia* L.), eucalyptus (*Eucalyptus globules* Labill.), camphor tree (*Cinnamomum camphora* L.), jujube (*Ziziphus lotus* L.), sage (*Salvia officinalis* L.), harmal (*Peganum harmala* L.), and multifloral honey. Table 1 provides detailed information on the type, region (GPS coordinates, climate), and botanical origins of the honey samples, including the scientific and common names of the plants that constitute their basic flora. All samples were collected between March 2017 and August 2018 and stored in sealed amber glass containers at 4 °C, protected from light and humidity. Previous studies have shown that honey remains stable under such conditions [18], and the samples exhibited no signs of fermentation, crystallisation, or sensory defects at the time of evaluation in 2025.

**Table 1:** Geographical origins of honey samples from western regions in Algeria.

Region	Sample	Flower type	Scientific name	Botanical family	Location	GPS coordinates	Climate	Altitude (m)	Harvest season/year
Tlemcen	S1	Lavender	*Lavandula vera* D.C.	Lamiaceae	Sidi Djillali	34° 28’ 00’‘ N 1° 34’ 60’‘ W	Subhumid	1470	Summer 2018
	S2	Rosemary	*Rosmarinus officinalis* L.	Lamiaceae	Sidi Djillali	34° 28’ 00’‘ N 1° 34’ 60’‘ W	Subhumid	1470	Spring 2018
	S3	Multifloral	Multifloral	–	Sidi Djillali	34° 28’ 00’‘ N 1° 34’ 60’‘ W	Subhumid	1470	Spring 2018
	S4	Multifloral	Multifloral	–	Sidi Djillali	34° 28’ 00’‘ N 1° 35’ 00’‘ W	Subhumid	1425	Summer 2017
	S5	Multifloral	Multifloral	–	El Aricha	34° 13’ 22“ N 1° 15’ 21” W	Subhumid	1270	Summer 2017
	S6	Sweet white mustard	*Sinapis alba* L.	Brassicaceae	Aïn Fezza	34° 52’ 45“ N 1° 14’ 18” W	Subhumid	846	Summer 2017
	S7	Thyme	*Thymus vulgaris* L.	Lamiaceae	Beni Snous	34° 38’ 35’‘ N 1° 33’ 41’‘ W	Subhumid	835	Spring 2018
	S8	Milk thistle	*Silybum marianum* (L.) Gaertn.	Asteraceae	Beni Snous	34° 38’ 35’‘ N 1° 33’ 41’‘ W	Subhumid	835	Summer 2018
	S9	Multifloral	Multifloral	–	Oued Chouly	34° 56’ 52’‘ N 1° 03’ 17’‘ W	Subhumid	705	Autumn 2017
	S10	Carob	*Ceratonia siliqua* L.	Fabaceae	Oued Chouly	34° 56’ 52’‘ N 1° 03’ 17’‘ N	Subhumid	705	Autumn 2017
	S11	Thyme	*Thymus vulgaris* L.	Lamiaceae	Beni Mester	34° 52’ 00’‘ N 1° 25’ 00’‘ W	Subhumid	697	Spring 2017
	S12	Carob	*Ceratonia siliqua* L.	Fabaceae	Béni Ghazli	34° 52’ 34’‘ N 1° 07’ 56’‘ W	Subhumid	624	Spring 2017
	S13	Multifloral	Multifloral	–	Oued es Safsâf	34° 55’ 60’‘ N 1° 18’ 00“ W	Subhumid	551	Summer 2018
	S14	Multifloral	Multifloral	–	Sebaa Chioukh	35° 09’ 50’‘ N 1° 21’ 27’‘ W	Subhumid	514	Spring 2017
	S15	Multifloral	Multifloral	–	Hennaya	34° 57’ 00’‘ N 1° 22’ 00’‘ W	Subhumid	429	Summer 2017
	S16	Orange	*Citrus sinensis* L.	Rutaceae	Remchi	35° 03’ 00’‘ N 1° 26’ 00’‘ W	Subhumid	213	Spring 2017
	S17	Multifloral	Multifloral	–	Honaïne	35° 10’ 35’‘ N 1° 39’ 18’‘ W	Subhumid	197	Spring 2018
	S18	Milk thistle	*Silybum marianum* (L.) Gaertn.	Asteraceae	Honaïne	35° 10’ 35’‘ N 1° 39’ 18’‘ W	Subhumid	197	Summer 2018
Ain-Temouchent	S19	Multifloral	Multifloral	–	Oulhaça El Gherarba	35° 13’ 00’‘ N 1° 31’ 00’‘ W	Subhumid	232	Spring 2018
	S20	Multifloral	Multifloral	–	Beni Ghanem	35° 15’ 16’‘ N 1° 25’ 38’‘ W	Subhumid	220	Summer 2018
	S21	Multifloral	Multifloral	–	Bouzedjar	35° 34’ 28“ N 1° 10’ 01” W	Subhumid	104	Spring 2018
Sidi Bel Abbes	S22	Euphorbia	*Euphorbia* L.	Euphorbiaceae	Ras El Ma	34° 29’ 51’‘ N 0° 49’ 10’‘ W	Semi-arid	1105	Spring 2017
	S23	Milk thistle	*Silybum marianum* (L.) Gaertn.	Asteraceae	Telagh	34° 47’ 06“ N 0° 32’ 40” W	Semi-arid	987	Spring 2017
	S24	Multifloral	Multifloral	–	Lamtâr	35° 04’ 14“ N 0° 47’ 53” W	Semi-arid	578	Spring 2017
	S25	Eucalyptus	*Eucalyptus globulus* Labill.	Myrtaceae	Sidi Brahim	35° 15’ 38“ N 0° 34’ 03” W	Semi-arid	432	Spring 2017
Mostaganem	S26	Camphor	*Cinnamomum camphora* L.	Lauraceae	Sidi Ali	36° 06’ 17“ N 0° 25’ 24” E	Semi-arid	216	Autumn 2017
	S27	Eucalyptus	*Eucalyptus globulus* Labill.	Myrtaceae	Mostaganem	35° 56’ 00“ N 0° 05’ 00” E	Semi-arid	104	Summer 2017
	S28	Orange	*Citrus sinensis* L.	Rutaceae	Bouguirat	35° 45’ 05“ N 0° 15’ 12” E	Semi-arid	66	Spring 2017
Mascara	S29	Rosemary	*Rosmarinus officinalis* L.	Lamiaceae	Djebel Stamboul	35° 23’ 00“ N 0° 09’ 00” E	Semi-arid	932	Spring 2017
Tiaret	S30	Multifloral	Multifloral	–	Tiaret	34° 55’ 00“ N 1° 34’ 60” E	Semi-arid	1189	Spring 2018
Naâma	S31	Multifloral	Multifloral	–	Aïn Sefra	32° 45’ 20“ N 0° 35’ 09” W	Arid	1073	Spring 2017
	S32	Jujube	*Ziziphus lotus* L.	Rhamnaceae	Aïn Sefra	32° 45’ 20“ N 0° 35’ 09” W	Arid	1073	Spring 2017
	S33	Jujube	*Ziziphus lotus* L.	Rhamnaceae	Aïn Ben Khelil	33° 17’ 25“ N 0° 45’ 51” W	Arid	1156	Spring 2017
	S34	Sage	*Salvia officinalis* L.	Lamiaceae	Naâma	33° 17’ 25“ N 0° 45’ 51” W	Arid	1031	Spring 2017
	S35	Harmal	*Peganum harmala* L.	Zygophyllaceae	Mecheria	33° 33’ 00“ N 0° 17’ 00” W	Arid	891	Spring 2017
Bechar	S36	Multifloral	Multifloral	–	Djebel Antar	31° 56’ 34“ N 1° 55’ 52” W	Arid	1953	Winter 2017
	S37	Sweet white mustard	*Sinapis alba* L.	Brassicaceae	Oued Zouzfana	32° 04 ‘01“ N 1° 14’ 27” W	Arid	830	Spring 2017

Additionally, in the sensory evaluation, four Polish honeys were used as reference samples (C38: multifloral honey; C39: multifloral honey; C40: heather honey; C41: buckwheat honey). The Polish honeys were purchased from a local health food store and held veterinary certification permitting sale (Producer: Pasieka Michał Rombel, Mińsk Mazowiecki, Poland). Parallel physicochemical analyses of Polish references were not feasible; literature values were used to provide a broader representative range (see Table 5 in section 3.).

### 2.3 Moisture content

The refractometric method was employed to determine the water content of each honey sample using an Abbe 5 refractometer (Bellingham + Stanley Ltd., Xylem UK). Optical refractive index measurements were corrected to a standard temperature of 20°C by applying a correction coefficient of 0.00023/°C. All measurements were performed in triplicate, and the results were compared against the Chataway table [19], which provides the moisture content of honey in percentage.

### 2.4 pH and free acidity

The pH of each honey sample was measured using an Adwa AD8000 pH meter (Szeged, Hungary). To determine free acidity, 10 g of honey was dissolved in 75 mL of distilled water in a 250 mL beaker, and the pH was measured by immersing the pH electrode into the solution. Free acidity was then determined by titrating this solution with 0.1 M NaOH until a pH of 8.3 was reached. Phenolphthalein was used as an indicator to detect the appearance of a persistent pink colour lasting approximately ten seconds. A blank test was performed using distilled water under the same conditions. All experiments were conducted in triplicate, and the results were expressed in milliequivalents (meq) per kilogram of honey, following the methods described by Bogdanov *et al.* [19].

### 2.5 Hydroxymethylfurfural content (HMF)

The HMF content was measured using a SHIMADZU UVmini-1240 spectrophotometer (Shimadzu Europa GmbH, Germany), following the method described by White (1979). This procedure involved measuring the absorbance of a clarified solution containing 10% (w/v) honey dissolved in distilled water, compared to a reference solution of the same honey in which the chromophore at *λ*max (284 nm) of HMF was destroyed by adding 0.1% sodium bisulfite. The HMF content was expressed in mg/kg of honey [19].

### 2.6 Electrical conductivity (EC)

Electrical conductivity was measured at 20.0°C using a WTW inoLab® Cond conductivity meter (WTW GmbH & Co. KG, Weilheim, Germany). The measurements were performed on a solution containing 20% (w/v) honey dissolved in demineralised distilled water. Results were expressed in millisiemens per centimetre (mS/cm) [19].

### 2.7 Specific rotation

The specific rotation (${\left[\alpha \right]}_{\text{D}}^{\text{2}\text{0}}$) or optical activity was measured at 20°C using an OPTIKA polarimeter (Science Italy). Measurements were conducted in a clear solution containing 10% (w/v) honey dissolved in distilled water. This parameter is associated with the carbohydrate content of the honey [19].

### 2.8 Proline content

Proline, the main amino acid in honey, was quantified spectrophotometrically [12,20]. One milliliter of formic acid and 1 mL of a 3% ninhydrin solution prepared in ethylene glycol monomethyl ether were added to three test tubes containing 0.5 mL of deionized water (blank), 0.5 mL of a 32 mg/L proline standard solution (standard), and 0.5 mL of a 5% honey dilution (sample). After the tubes were sealed, the contents were mixed thoroughly and heated in a boiling water bath for 15 min, followed by 10 min in a water bath at 70 °C. Subsequently, 5 mL of a 50% 2-propanol–water solution was added to each tube 45 min after removal from the water bath, and the absorbance at a wavelength of 510 nm, measured using an OPTIZEN™ POP UV-Visible spectrophotometer (Mecasys Co., Ltd., South Korea), was recorded. The proline content was calculated using the formula (1) below, recommended by the European Commission’s Harmonised Methods for Honey, and expressed in mg/kg of honey:


$$Proline\;\left(\frac{mg}{kg}\right)=\left(\frac{E_{S}}{E_{a}}\right)\;x\;\left(\frac{E_{\text{1}}}{E_{\text{2}}}\right)\;x\;\text{8}\text{0}$$ (1)

where $E_{s}$ represents the absorbance of the honey sample solution, $E_{a}$ is the absorbance of the proline standard solution (average of two measurements), $E_{\text{1}}$ denotes the mass of proline used for the stock solution (mg), $E_{\text{2}}$ is the weight of the honey sample (g), and 80 corresponds to the dilution factor.

### 2.9 Honey colour

The instrumental colour measurement was performed using an Envi Sense NH310 colourimeter. The measurement system employed was the CIELAB system, where L* represents brightness as a spatial vector, while a* and b* are trichromatic coordinates. Positive a* values correspond to red, negative values to green; positive b* values correspond to yellow, and negative values to blue. Additionally, the CIE L*, $C_{ab}^{*}$, and $h_{a}^{b^{\circ }}$ values were presented, where $C_{ab}^{*}$ denotes chroma and $h_{a}^{b^{\circ }}$ represents the hue angle. These parameters were derived from the a* and b* values on the CIE Lab* scale, with the L* value remaining consistent across scales. The diameter of the measurement aperture was 8 mm. Each test was measured in quadruplicate.

### 2.10 Sugar content

To determine the sugar composition, 1 g of honey was weighed in duplicate into 50 mL graduated flasks. The flasks were filled to volume with distilled water, mixed thoroughly, and filtered. The filtrate was analysed using a Gilson HPLC system equipped with Gilson 306 pumps, a 234 Autoinjector automatic sampler, an amine column (Aminex HPX-87H, BioRad, 300 × 7.8 mm), and a refractive index detector (Knauer K2300). The mobile phase consisted of 0.03 M sulphuric acid (VI), and the separation process was performed at 42°C using a Phenomenex Thermasphere TS130 thermostat, with a flow rate of 0.5 mL/min. Quantitative analysis was conducted using previously determined calibration curves for aqueous solutions of glucose, fructose, maltose, and sucrose, in the range of 0.02 to 20 mg/mL.

### 2.11 Sensory analysis

The sensory analysis was carried out at the Department of Food Quality Analysis and Evaluation, Sensory Analysis Laboratory, University of Life Sciences in Lublin. The sensory panel comprised nine trained assessors certified according to ISO 8586 [21]. The panel evaluated the samples using two complementary methods: (1) a five-point hedonic scale (+2 = “like very much” … –2 = “dislike very much”) for taste, aroma, and colour; and (2) a Check-All-That-Apply (CATA) questionnaire including 11 descriptors (five odour descriptors: weak aroma, strong aroma, very strong aroma, herbal, characteristic herbal “individual description”; six taste descriptors: sweet, mild, sharp-scratching, bitter, herbal, characteristic herbal “individual description”).

Sample preparation and testing procedure:

Honey samples were prepared following a modified version of the protocol described by Moumeh*et al.* [22]. Algerian honeys were coded as S1–S37 (Table 1), and Polish reference honeys as C38 (multifloral), C39 (multifloral), C40 (heather), and C41 (buckwheat). Each sample (30 g) was presented in a 100 mL transparent jar at 20 ± 2 °C. Every sample was evaluated in three separate sessions. To enhance initial olfactory perception, assessors could apply the honey to the jar walls using a spatula. A bowl of coffee beans was provided to each assessor for nasal palate cleansing. For taste and retronasal aroma assessment, a small amount of honey was placed on the tongue with a disposable spatula and allowed to dissolve for a few seconds without inhalation; subsequent aroma release was performed by exhaling through the nose while the sample remained on the palate. Palate cleansing with water was provided between samples.

This study was conducted in accordance with the Declaration of Helsinki (October 2013, Fortaleza, Brazil), and the protocol was approved by the Ethics Committee of the University of Life Sciences in Lublin (Approval No. UKE/54/2025). Written informed consent was obtained from all panelists; the ethics committee approved the consent procedure. Recruitment period: Panelist recruitment was conducted on 01/07/2025 (start and end), and the sensory evaluation was carried out between 01/07/2025 and 03/07/2025.

### 2.12 Statistical analysis

Statistical analyses were carried out using SPSS software (version 22.0). All experiments were performed in triplicate, and the results were expressed as means ± standard deviation (SD). Differences between samples were assessed through one-way ANOVA, followed by Duncan’s multiple range test to compare the means at a significance threshold of *p* = 0.05.

Data Hedonic scores for taste, aroma and colour were expressed as means ± SD and compared between Algerian and Polish honeys by independent two-sample t-tests (α = 0.05). Check-All-That-Apply (CATA) responses were converted into attribute-selection frequencies and analysed by Cochran’s Q test to detect overall differences among sample groups; post-hoc pairwise comparisons were performed using McNemar’s test with Bonferroni correction. Multivariate analysis of sensory descriptors was conducted by Principal Component Analysis (PCA) on the correlation matrix of standardized CATA-frequency variables and mean hedonic scores (SPSS v.22). To explore sensory-based sample grouping, hierarchical cluster analysis (HCA) was applied to the first two principal-component scores using Ward’s method and squared Euclidean distances. All tests were two-tailed, and statistical significance was set at **p* *< 0.05.

## 3. Results and discussion

### 3.1 Sample origin

Thirty-seven honey samples (S1–S37) of diverse floral origins were collected from beekeepers in western Algeria (Tlemcen, Ain-Temouchent, Sidi Bel Abbes, Mostaganem, Mascara, Tiaret, Naâma, Bechar), regions marked by contrasting topography, climate, and ecology. Table 2 summarises their physicochemical parameters.

**Table 2:** Physical and chemical parameters of the analyzed honeys.

Sample	Moisture content * (%)	pH *	Free acidity * (meq/kg)	EC * (mS/cm)	HMF * (mg/kg)	Proline * (mg/kg)	${\left[\alpha \right]}_{𝐃}^{20}$ *
S1	17.33 ± 0.30	3.61 ± 0.02	9.67 ± 1.15	0.45 ± 0.01	11.27 ± 1.51	293.31 ± 0.84	(-) 12.50 ± 0.25
S2	17.13 ± 0.11	3.47 ± 0.15	12.50 ± 0.87	0.26 ± 0.00	32.26 ± 0.91	431.32 ± 5.32	(-) 10.89 ± 0.03
S3	18.27 ± 0.23	3.89 ± 0.03	14.00 ± 1.00	0.21 ± 0.00	6.30 ± 0.65	398.73 ± 0.18	(-) 9.55 ± 0.20
S4	17.43 ± 0.15	4.85 ± 0.18	28.33 ± 1.52	0.27 ± 0.01	25.70 ± 1.06	587.93 ± 0.73	(-) 9.92 ± 0.06
S5	15.46 ± 0.11	5.60 ± 0.04	13.00 ± 0.87	0.25 ± 0.00	47.43 ± 2.22	265.95 ± 1.28	(-) 8.85 ± 0.03
S6	18.60 ± 0.00	4.00 ± 0.03	18.67 ± 1.53	0.55 ± 0.00	33.93 ± 2.94	987.08 ± 2.61	(-) 14.08 ± 0.04
S7	20.87 ± 0.61	4.29 ± 0.02	17.33 ± 0.58	0.29 ± 0.00	9.73 ± 0.93	421.04 ± 4.32	(-) 10.39 ± 0.25
S8	17.07 ± 0.11	4.29 ± 0.04	11.00 ± 1.00	0.47 ± 0.01	7.63 ± 1.98	725.14 ± 6.43	(-) 11.32 ± 0.21
S9	16.80 ± 0.40	4.66 ± 0.03	22.67 ± 1.15	0.25 ± 0.02	3.82 ± 0.59	655.21 ± 0.27	(-) 11.17 ± 0.01
S10	18.40 ± 0.00	4.39 ± 0.19	15.83 ± 1.04	0.42 ± 0.01	26.55 ± 4.23	800.42 ± 2.75	(-) 8.87 ± 0.02
S11	15.93 ± 0.30	4.62 ± 0.03	30.33 ± 1.53	0.44 ± 0.01	19.76 ± 0.32	924.54 ± 6.14	(-) 11.02 ± 0.02
S12	18.73 ± 0.30	4.37 ± 0.02	8.00 ± 1.00	0.72 ± 0.02	40.62 ± 4.41	684.42 ± 2.57	(-) 11.40 ± 0.01
S13	15.13 ± 0.11	4.11 ± 0.01	20.00 ± 1.00	0.91 ± 0.01	17.81 ± 0.98	734.81 ± 2.23	(-) 10.37 ± 0.09
S14	18.07 ± 0.30	4.61 ± 0.05	11.00 ± 1.00	0.51 ± 0.01	6.02 ± 0.82	359.58 ± 1.76	(-) 10.59 ± 0.07
S15	16.13 ± 0.41	4.29 ± 0.02	21.33 ± 1.53	0.38 ± 0.00	8.42 ± 3.74	478.75 ± 2.87	(-) 9.17 ± 0.01
S16	18.00 ± 0.00	4.27 ± 0.01	15.00 ± 1.00	0.16 ± 0.01	49.43 ± 0.72	412.83 ± 4.41	(-) 8.75 ± 0.02
S17	20.06 ± 0.92	4.63 ± 0.04	19.33 ± 1.52	0.42 ± 0.00	4.64 ± 0.80	871.39 ± 1.06	(-) 11.66 ± 0.07
S18	16.67 ± 0.11	3.81 ± 0.09	15.67 ± 1.15	0.32 ± 0.01	35.63 ± 0.42	905.82 ± 3.13	(-) 10.69 ± 0.16
S19	19.87 ± 0.41	5.10 ± 0.06	39.33 ± 1.15	1.18 ± 0.02	7.91 ± 0.51	301.81 ± 0.90	(-) 10.48 ± 0.02
S20	16.33 ± 0.23	4.38 ± 0.02	10.33 ± 0.11	1.04 ± 0.01	2.45 ± 0.34	1006.34 ± 3.78	(-) 10.92 ± 0.01
S21	18.06 ± 0.23	4.19 ± 0.01	11.83 ± 0.29	0.50 ± 0.00	9.48 ± 1.73	899.21 ± 2.61	(-) 9.86 ± 0.02
S22	14.87 ± 0.30	4.36 ± 0.53	15.67 ± 1.15	0.24 ± 0.00	4.44 ± 0.82	458.30 ± 2.19	(-) 9.51 ± 0.04
S23	16.66 ± 0.11	4.52 ± 0.43	20.33 ± 1.52	0.44 ± 0.01	7.54 ± 0.64	1132.73 ± 2.49	(-) 10.72 ± 0.02
S24	17.60 ± 0.00	3.96 ± 0.15	20.67 ± 3.05	0.16 ± 0.00	19.31 ± 0.89	794.23 ± 2.37	(-) 9.70 ± 0.03
S25	17.13 ± 0.11	4.10 ± 0.01	20.33 ± 2.31	0.32 ± 0.00	18.11 ± 3.17	1078.64 ± 8.87	(-) 10.46 ± 0.20
S26	18.07 ± 0.11	4.72 ± 0.03	25.00 ± 2.00	0.32 ± 0.00	4.66 ± 0.79	491.47 ± 7.21	(-) 9.46 ± 0.04
S27	16.13 ± 0.11	4.29 ± 0.02	33.67 ± 1.53	0.58 ± 0.01	10.47 ± 2.35	741.25 ± 2.06	(-) 11.35 ± 0.03
S28	17.47 ± 0.23	4.65 ± 0.01	28.00 ± 2.00	0.21 ± 0.00	16.77 ± 0.64	285.59 ± 0.88	(-) 10.13 ± 0.02
S29	16.80 ± 0.00	4.21 ± 0.02	40.33 ± 2.52	0.25 ± 0.00	34.90 ± 2.54	1200.66 ± 1.92	(-) 10.56 ± 0.02
S30	15.87 ± 0.30	4.01 ± 0.02	9.33 ± 0.76	0.36 ± 0.00	1.79 ± 0.02	1117.33 ± 3.63	(-) 8.30 ± 0.14
S31	16.33 ± 0.23	3.94 ± 0.01	17.16 ± 0.76	0.44 ± 0.00	4.34 ± 0.26	326.50 ± 1.05	(-) 9.57 ± 0.20
S32	15.80 ± 0.40	4.69 ± 0.01	17.16 ± 1.52	0.25 ± 0.00	28.29 ± 0.37	478.67 ± 0.77	(-) 10.40 ± 0.03
S33	15.27 ± 0.30	5.31 ± 0.01	12.33 ± 1.52	0.22 ± 0.00	15.25 ± 3.75	637.61 ± 1.35	(-) 10.10 ± 0.02
S34	14.67 ± 0.11	4.53 ± 0.07	22.67 ± 2.08	0.20 ± 0.00	36.53 ± 1.27	508.88 ± 1.78	(-) 10.96 ± 0.03
S35	16.60 ± 0.20	4.99 ± 0.01	15.83 ± 0.76	0.24 ± 0.00	45.36 ± 1.43	394.83 ± 0.63	(-) 10.98 ± 0.02
S36	18.20 ± 0.53	5.01 ± 0.04	9.00 ± 1.00	0.46 ± 0.00	4.54 ± 0.53	279.72 ± 2.74	(-) 10.59 ± 0.33
S37	16.93 ± 0.11	4.48 ± 0.06	13.83 ± 1.26	0.25 ± 0.00	40.64 ± 0.63	845.61 ± 4.48	(-) 10.22 ± 0.01
Statistics							
Mean	17.02	4.35	15.29	0.41	14.54	678.46	−10.62
SD	0.26	0.08	1.03	0.00	1.12	2.97	0.10

All values are expressed as means of triplicate determinations ± SD. EC: Electrical conductivity; HMF: Hydroxymethyl furfural; ${\left[\alpha \right]}_{\text{D}}^{\text{2}\text{0}}$: Specific optical rotation. *: The results are statistically significant at **p* *< 0.05; in the same column, the values are significantly different at *p* < 0.001.

### 3.2 Moisture as an indicator of stability

Moisture is a key determinant of honey maturity, fermentation risk, and crystallisation. Values ranged from 14.67 ± 0.11% to 20.87 ± 0.61%. The highest were thyme (S7: 20.87 ± 0.61%) and multifloral (S17: 20.06 ± 0.92%) honeys from Tlemcen, while the lowest were sage from Naâma (S34: 14.67 ± 0.11%) and Euphorbia from Sidi Bel Abbes (S22: 14.87 ± 0.30%).

Algerian eucalyptus honeys (S25: 17.13 ± 0.11%, S27: 16.13 ± 0.11%) had lower values than Moroccan (19.8 ± 0.1%) [23] and Tunisian honeys (19.12 ± 0.07%) [24]. Carob honeys (S10: 18.40 ± 0.00%, S12: 18.73 ± 0.30%) were also below Moroccan carob (20.0 ± 0.10%) [23]. Conversely, rosemary honeys (S2: 17.13 ± 0.11%, S4: 17.43 ± 0.15%) and multifloral (S3: 18.27 ± 0.23%) were comparable with Tunisian rosemary (17.27 ± 0.01%) [24], Moroccan multifloral (17.8 ± 0.10%) [23], and Turkish multifloral (18.39 ± 1.30%) [25].

Overall, 12 samples exceeded 18%, a threshold linked to fermentation [26], while two Tlemcen honeys (S7, S17) surpassed the Codex Alimentarius limit of 20%, likely reflecting harvest or handling issues [27]. Conversely, two samples had < 15%, making them overly viscous and prone to crystallisation. Although techniques like pasteurisation or dehydration are sometimes used to adjust honey’s moisture, these methods are not always accepted as they can alter the honey’s natural state [28].

Regional trends were evident: honeys from drier zones averaged 16.47 ± 0.20%, versus 18.00 ± 0.36% in more humid areas, confirming climatic influence [29]. Botanical origin also played a decisive role [30]. The most humid were from carob (18.56 ± 0.15%; S10, S12), thyme (18.40 ± 0.45%; S7, S11), orange (18.10 ± 0.26%; S16, S28), and camphor (18.07 ± 0.11%; S26). Intermediate levels were recorded for sweet white mustard (17.76 ± 0.05%; S6, S37), lavender (17.33 ± 0.30%; S1), rosemary (16.96 ± 0.05%; S2, S29), milk thistle (16.80 ± 0.11%; S8, S18, S23), eucalyptus (16.63 ± 0.11%; S25, S27), and harmal (16.60 ± 0.20%; S35). The driest were jujube (15.53 ± 0.35%; S32, S33), spurge (14.87 ± 0.30%; S22), and sage (14.67 ± 0.11%; S34).

### 3.3 Acidity profiles and pH balance

Acidity, resulting from more than 30 organic acids of floral and bee origin, strongly influences honey’s flavour, colour, and resistance to microbial spoilage. Predominant acids can serve as markers of floral origin, with darker honeys generally displaying higher acidity. pH also differentiates nectar honeys (pH 3.5–4.5) from honeydew honeys (pH 4.5–5.5), regardless of geographical origin.

Measured pH values ranged from 3.47 ± 0.15 (S2) to 5.60 ± 0.04 (S5) (Table 2), fully complying with Codex Alimentarius standards [31]. These results align with previously reported ranges for Algerian honeys (3.75–5.56 [32]; 3.6 ± 0.16–6.2 ± 0.30 [33]; 3.5–4.7 [34]) and are comparable to those documented for honeys from Morocco (4.17 ± 0.05–5.05 ± 0.13 [35]), Tunisia (3.45–4.63 [24]), Palestine (3.03–5.98 [36]), and Spain (4.4 ± 0.20 [37]).

pH variation was clearly linked to floral origin [30]. The most acidic honeys were rosemary (3.47 ± 0.15; S2) and lavender (3.61 ± 0.02; S1), followed by white mustard (4.00 ± 0.03; S6), eucalyptus (4.10 ± 0.01; S25), orange (4.27 ± 0.01; S16), thyme (4.29 ± 0.02; S7), Euphorbia (4.36 ± 0.53; S22), and carob (4.39 ± 0.19; S10). In addition to floral origin, geographical factors, soil type, and mineral content may also contribute to differences in pH [38].

Free acidity ranged from 8.00 ± 1.00 (S12) to 40.33 ± 2.52 meq/kg (S29) (Table 2), all below the Codex Alimentarius maximum of 50 meq/kg [31]. These results align with Algerian honeys (14.1–46.5 meq/kg [39]), Moroccan honeys (11.0 ± 2.29–42.50 ± 2.29 meq/kg [35]), Tunisian honeys (7.11 ± 0.20–27.20 ± 0.20 meq/kg), and Turkish honeys (8.0–46.89 meq/kg) [40]. Variations can be attributed to botanical origin and harvest season [40]. Importantly, none of the samples exceeded regulatory limits, confirming their freshness and high quality.

### 3.4 Electrical conductivity and mineral signature

Electrical conductivity (EC) is a key metric for differentiating between nectar and honeydew honeys. Honeydew honeys typically have a higher mineral content, resulting in a greater EC. Furthermore, darker honeys generally exhibit more efficient electrical conductivity [5,41].

Observed EC values ranged from 0.16 ± 0.01 to 1.18 ± 0.02 mS/cm (Table 2). With the exception of multifloral honeys (S13, S19, S20) that exceeded 0.8 mS/cm, the majority were classified as nectar honeys (EC ≤ 0.8 mS/cm). All samples satisfied the Codex Alimentarius standards [31].

These results agree with previously reported ranges for Algerian honeys (0.29–1.35 mS/cm [39]; 0.133–1.460 mS/cm [34]) and are consistent with values documented for Tunisian honeys (0.39 ± 0.02–0.89 ± 0.06 mS/cm [24]), Turkish honeys (0.19 ± 0.06–1.13 ± 0.25 mS/cm [25]), and Moroccan honeys (0.36 ± 0.02–1.35 ± 0.03 mS/cm [35]). Elevated EC values are generally associated with darker or pollen-rich honeys containing higher concentrations of ionisable minerals, organic acids, and proteins, and may also reflect the presence of both nectar and honeydew components [31].

The floral origin of the honey samples clearly influenced their EC, as reported in earlier studies [30,38,41,42]. The highest values were observed in multifloral honeys (0.91 ± 0.01 mS/cm, S13; 1.18 ± 0.02 mS/cm, S19; 1.04 ± 0.01 mS/cm, S20), followed by carob (0.72 ± 0.02 mS/cm, S12), eucalyptus (0.58 ± 0.01 mS/cm, S27), sweet white mustard (0.55 ± 0.00 mS/cm, S6), milk thistle (0.47 ± 0.01 mS/cm, S8), lavender (0.45 ± 0.01 mS/cm, S1), and thyme (0.44 ± 0.01 mS/cm, S11). In contrast, lower EC values were recorded for camphor (0.32 ± 0.00 mS/cm, S26), rosemary (0.26 ± 0.00 mS/cm, S2), jujube (0.25 ± 0.00 mS/cm, S32), euphorbia (0.24 ± 0.00 mS/cm, S22), harmal (0.24 ± 0.00 mS/cm, S35), orange (0.21 ± 0.00 mS/cm, S28), and sage (0.20 ± 0.00 mS/cm, S34).

Such variability is a robust indicator, directly influenced by the concentration of dissolved components like mineral salts, organic acids, and proteins [43]. The levels of these constituents are, in turn, determined by the honey’s botanical origin and the specific regional climatic conditions of its production [44], which dictate its overall chemical composition.

### 3.5 HMF levels and honey freshness

Hydroxymethylfurfural (HMF), a sugar degradation product, is widely recognised as an indicator of honey freshness and heat exposure. Quantification by UV spectrophotometry (White’s method) yielded values between 1.79 ± 0.02 and 49.43 ± 0.72 mg/kg (Table 2).

Five samples (S5, S12, S16, S35, and S37) exceeded the Codex Alimentarius limit of 40 mg/kg, recording 47.43 ± 2.22, 40.62 ± 4.41, 49.43 ± 0.72, 45.36 ± 1.43, and 40.64 ± 0.63 mg/kg, respectively. However, all remained below the threshold of 60 mg/kg set for honeys from tropical or warm climates, confirming compliance with international standards. Elevated levels in S12 and S16 may reflect heat exposure or inadequate handling prior to storage, while high values in S5, S35, and S37 likely result from the semi-arid and arid climates of El Aricha, Mecheria, and Bechar. In contrast, 18 samples had HMF levels below 15 mg/kg, demonstrating excellent freshness.

Marked variation was observed by honey type. The lowest values were found in multifloral honeys (1.79 ± 0.02 mg/kg, S30; 2.45 ± 0.34 mg/kg, S20), euphorbia honey (4.44 ± 0.82 mg/kg, S22), and camphor honey (4.66 ± 0.79 mg/kg, S26), while the highest were recorded in orange honey (49.43 ± 0.72 mg/kg, S16) and harmal honey (45.36 ± 1.43 mg/kg, S35). These findings emphasise that HMF levels depend more on processing, storage, and climatic conditions than floral origin.

The results were slightly higher than those previously reported for Algerian honeys (8.80–39.62 mg/kg [45]; 1.30–31.8 mg/kg [39]), Tunisian honeys (12.07 ± 1.00–27.43 ± 1.50 mg/kg [24]), Portuguese honeys (7.4 ± 0.10–28.4 ± 0.10 mg/kg [46]), and Turkish honeys (1.59 ± 1.32–11.83 ± 4.17 mg/kg [25]). Conversely, they were lower than those reported for other Algerian (11.04 ± 0.66–82.00 ± 0.58 mg/kg [47]; 2.84–117.7 mg/kg [48]) and Palestinian honeys (10.16 ± 0.53–81.86 ± 2.64 mg/kg [49]).

Overall, while some samples exhibited elevated HMF, all complied with international standards, confirming their freshness and acceptable quality.

### 3.6 Proline as a marker of maturity

Amino acids constitute about 1.0% of honey, with proline 50–85%. The 26 amino acids vary with origin (nectar or honeydew), and proline declines during storage; it is an indicator of maturity and authenticity. International standards require ≥180 mg/kg. In Table 2, proline ranged from 265.95 ± 1.28 to 1200.66 ± 1.92 mg/kg. Among floral honeys, orange blossom (285.59 ± 0.88 mg/kg, S28) had the lowest level, followed by lavender (293.31 ± 0.84 mg/kg, S1), harmal (394.83 ± 0.63 mg/kg, S35), thyme (421.04 ± 4.32 mg/kg, S7), euphorbia (458.30 ± 2.19 mg/kg, S22), jujube (478.67 ± 0.77 mg/kg, S32), and camphor (491.47 ± 7.21 mg/kg, S26). The highest values were rosemary (1200.66 ± 1.92 mg/kg, S29) and milk thistle (1132.73 ± 2.49 mg/kg). Several exceeded 500 mg/kg, reflecting nectar–pollen ratios. Concentrations matched Moroccan honeys (441.67–1207.55 mg/kg) [50] and Spanish honeys (510 ± 216–1322 mg/kg) [51], and were higher than Algerian (551.88 ± 10.50–852.0 ± 9.90 mg/kg) [47], Palestinian (229.44 ± 3.24–720.87 ± 5.18 mg/kg) [49], Turkish (357.00 ± 34.38–758.56 ± 67.73 mg/kg) [25], and Portuguese honeys (412.3 ± 4.8–566.6 ± 4.8 mg/kg) [46]. All samples exceeded 180 mg/kg, supporting maturity and purity; variation across and within floral sources reflects botanical origin.

### 3.7 Optical rotation

Honey optical rotation depends on its sugar composition and concentration. In several countries, such as Greece, Italy, and the United Kingdom, it is used to distinguish floral honeys (levorotatory) from honeydew honeys (dextrorotatory). Dextrorotation in honeydew is linked to lower fructose and higher di- and oligosaccharides.

All analysed samples exhibited negative values, including honeydew honeys (Table 2). The most negative was sweet white mustard honey (−14.08 ± 0.04°, S6), followed by lavender honey (−12.50 ± 0.25°, S1). The least negative were multifloral (−8.30 ± 0.14°, S30; −8.85 ± 0.03°, S5), orange (−8.75 ± 0.02°, S16), and carob honeys (−8.87 ± 0.02°, S10). These results agree with Algerian honeys (−14.35 ± 0.03° to −4.65 ± 0.03°) [52], (−18.46 ± 0.76° to −2.07 ± 0.24°) [53], Polish buckwheat (−12.0 ± 1° to −7.5 ± 0.5°) [54], Portuguese honeys (−15.4 ± 0.6° to −11.9 ± 0.8°) [46], and Spanish honeys (−8.94° to −14.13°) [55].

Optical rotation may thus support monofloral discrimination. Its simplicity, speed, and cost-effectiveness warrant consideration for standardised international application.

### 3.8 Sugars and crystallisation potential

Differentiation between pure and adulterated honey is primarily based on sugar composition. Natural honey generally contains ~40% fructose and ~30% glucose, with sucrose usually <5%, except in specific floral varieties [56].

Four sugars—fructose, glucose, sucrose, and maltose—were quantified. Total sugar content ranged from 68.12 ± 0.55% (S35, harmal) to 81.47 ± 0.05% (S20, multifloral) (Table 3). Fructose exceeded glucose in all but two samples, confirming authenticity. The highest fructose concentration was found in multifloral honey (47.47 ± 1.21%, S31), whereas the lowest occurred in harmal honey (33.88 ± 0.34%, S35). Glucose values ranged from 22.07 ± 0.17% (S4, multifloral) to 38.01 ± 0.90% (S37, sweet white mustard). Notably, euphorbia (S22) and sweet white mustard (S37) honeys contained more glucose than fructose, a feature also observed in rapeseed (*Brassica napus*) and dandelion (*Taraxacum officinale*) honeys [57].

**Table 3:** Sugar content analysis results.

Sample	G * (%)	F * (%)	(M + S) * (%)	(F + G) * (%)	F/G *	G/W *	Total sugar content *(%)
S1	31.48 ± 0.24	38.94 ± 0.47	5.94 ± 0.22	70.42 ± 0.71	1.23 ± 0.00	1.83 ± 0.04	76.37 ± 0.93
S2	30.65 ± 1.42	40.36 ± 0.40	7.11 ± 0.74	71.01 ± 1.82	1.31 ± 0.05	1.79 ± 0.07	78.13 ± 2.56
S3	32.40 ± 0.20	40.76 ± 0.79	6.67 ± 0.09	73.16 ± 0.99	1.25 ± 0.02	1.78 ± 0.02	79.84 ± 1.08
S4	22.07 ± 0.17	43.53 ± 0.14	7.75 ± 0.22	65.60 ± 0.03	1.97 ± 0.02	1.27 ± 0.01	73.35 ± 0.26
S5	34.64 ± 0.46	34.12 ± 0.15	6.39 ± 0.16	68.76 ± 0.61	0.98 ± 0.00	2.25 ± 0.03	75.15 ± 0.76
S6	32.13 ± 0.00	36.22 ± 0.28	2.24 ± 0.03	68.34 ± 0.28	1.12 ± 0.00	1.73 ± 0.00	70.59 ± 0.25
S7	30.01 ± 0.34	37.24 ± 0.65	8.14 ± 0.01	67.26 ± 0.99	1.24 ± 0.01	1.42 ± 0.00	75.40 ± 0.98
S8	31.98 ± 0.06	41.50 ± 2.06	3.76 ± 0.01	73.49 ± 2.12	1.29 ± 0.06	1.87 ± 0.02	77.24 ± 2.11
S9	34.33 ± 0.08	37.88 ± 0.09	5.21 ± 0.05	72.21 ± 0.18	1.10 ± 0.00	2.07 ± 0.04	77.43 ± 0.23
S10	31.10 ± 0.07	41.65 ± 0.31	5.33 ± 0.05	72.76 ± 0.38	1.33 ± 0.00	1.69 ± 0.01	78.09 ± 0.44
S11	33.99 ± 2.42	40.31 ± 2.07	4.54 ± 0.06	74.30 ± 4.50	1.18 ± 0.02	2.11 ± 0.13	78.84 ± 4.43
S12	34.32 ± 0.09	35.87 ± 0.04	4.11 ± 0.06	70.20 ± 0.05	1.04 ± 0.00	1.85 ± 0.03	74.31 ± 0.11
S13	33.07 ± 0.13	39.08 ± 0.51	3.91 ± 0.07	72.15 ± 0.64	1.18 ± 0.01	2.19 ± 0.03	76.06 ± 0.71
S14	30.25 ± 0.07	41.23 ± 0.48	3.87 ± 0.83	71.49 ± 0.54	1.36 ± 0.01	1.66 ± 0.02	75.36 ± 1.38
S15	28.31 ± 2.63	39.91 ± 1.62	5.66 ± 0.16	68.22 ± 4.26	1.41 ± 0.07	1.78 ± 0.15	73.88 ± 4.42
S16	28.26 ± 2.69	39.85 ± 1.69	5.60 ± 0.23	68.11 ± 4.38	1.41 ± 0.08	1.57 ± 0.15	73.71 ± 4.61
S17	35.27 ± 0.87	38.77 ± 0.68	4.22 ± 0.02	74.04 ± 1.55	1.09 ± 0.00	1.71 ± 0.04	78.26 ± 1.52
S18	34.67 ± 0.35	39.93 ± 0.82	4.19 ± 0.09	74.61 ± 1.17	1.15 ± 0.01	2.08 ± 0.01	78.80 ± 1.26
S19	30.81 ± 0.00	37.53 ± 0.07	6.23 ± 0.01	68.35 ± 0.06	1.22 ± 0.00	1.53 ± 0.01	74.58 ± 0.05
S20	37.17 ± 0.02	40.78 ± 0.09	3.53 ± 0.02	77.94 ± 0.08	1.09 ± 0.00	2.29 ± 0.00	81.47 ± 0.05
S21	31.92 ± 0.61	37.34 ± 1.34	4.93 ± 0.17	69.27 ± 1.95	1.17 ± 0.01	1.75 ± 0.03	74.20 ± 1.78
S22	36.99 ± 0.14	35.85 ± 0.01	5.33 ± 0.09	72.84 ± 0.12	0.97 ± 0.00	2.52 ± 0.02	78.18 ± 0.21
S23	28.84 ± 0.25	39.15 ± 0.27	5.11 ± 0.01	67.99 ± 0.52	1.35 ± 0.00	1.73 ± 0.00	73.10 ± 0.53
S24	27.87 ± 0.06	38.67 ± 0.23	4.64 ± 0.01	66.53 ± 0.29	1.38 ± 0.00	1.58 ± 0.01	71.17 ± 0.28
S25	27.87 ± 0.06	38.67 ± 0.23	4.64 ± 0.01	66.53 ± 0.29	1.38 ± 0.01	1.63 ± 0.01	71.17 ± 0.28
S26	30.03 ± 0.62	43.13 ± 0.97	6.08 ± 0.00	73.16 ± 0.35	1.43 ± 0.06	1.66 ± 0.02	79.24 ± 0.35
S27	30.53 ± 0.48	37.45 ± 0.40	4.09 ± 0.01	67.98 ± 0.88	1.22 ± 0.01	1.90 ± 0.01	72.08 ± 0.90
S28	31.16 ± 0.08	37.91 ± 0.12	7.02 ± 0.06	69.07 ± 0.04	1.21 ± 0.01	1.79 ± 0.03	76.09 ± 0.03
S29	31.45 ± 0.18	41.27 ± 0.11	5.90 ± 0.16	72.72 ± 0.29	1.31 ± 0.00	1.87 ± 0.01	78.62 ± 0.45
S30	35.60 ± 0.01	40.13 ± 0.07	5.12 ± 0.79	75.73 ± 0.08	1.13 ± 0.00	2.27 ± 0.02	80.85 ± 0.88
S31	27.16 ± 0.71	47.47 ± 1.21	6.24 ± 0.09	74.63 ± 1.92	1.75 ± 0.00	1.68 ± 0.04	80.87 ± 2.01
S32	33.04 ± 0.06	39.78 ± 0.25	5.88 ± 0.02	72.82 ± 0.31	1.20 ± 0.01	2.07 ± 0.04	78.70 ± 0.33
S33	30.96 ± 0.04	37.53 ± 0.01	6.62 ± 0.13	68.50 ± 0.04	1.21 ± 0.00	2.01 ± 0.03	75.12 ± 0.17
S34	34.50 ± 0.27	36.38 ± 0.06	5.97 ± 0.18	70.89 ± 0.21	1.05 ± 0.01	2.35 ± 0.01	76.86 ± 0.39
S35	27.83 ± 0.11	33.88 ± 0.34	6.41 ± 0.10	61.71 ± 0.45	1.21 ± 0.01	1.67 ± 0.02	68.12 ± 0.55
S36	30.53 ± 0.03	35.39 ± 0.10	8.22 ± 0.02	65.92 ± 0.07	1.16 ± 0.00	1.71 ± 0.01	74.14 ± 0.05
S37	38.01 ± 0.90	34.45 ± 0.12	3.82 ± 0.16	72.47 ± 1.03	0.90 ± 0.02	2.24 ± 0.05	76.29 ± 1.19
Statistics							
Mean	31.35	38.52	5.57	70.66	1.22	1.85	75.89
SD	0.43	0.59	0.15	0.90	0.01	0.03	1.15

All values are expressed as means of triplicate determinations ± SD. G: Glucose; F: Fructose; M: Maltose; S: Sucrose; W: Water; *: The results indicate statistical significance at **p* *< 0.05.

Sucrose and maltose were detected in all samples, ranging from 2.24 ± 0.03% (S6) to 8.22 ± 0.02% (S36), averaging 5.41%. Values remained below the 5% limit for sucrose in pure honey, confirming absence of adulteration. Maltose was always <30 mg/g, in agreement with typical natural profiles [58].

Crystallisation tendencies were assessed using the fructose-to-glucose (F/G) and glucose-to-water (G/W) ratios [57,59]. The F/G ratio ranged from 0.90 ± 0.02 (S37) to 1.97 ± 0.02 (S4), with an overall mean of 1.24. Three samples (S37, S22, S5) had ratios <1.0, indicating rapid crystallisation potential [27,56]. Conversely, samples S4 (1.97 ± 0.02) and S31 (1.75 ± 0.00) exhibited fructose predominance and a low glycaemic index, making them beneficial for individuals with impaired glucose tolerance [60].

The G/W ratio, more predictive of crystallization [57,61], exceeded 2.0 in one-third of the samples, consistent with their low moisture levels. None fell below 1.0, indicating stability. Crystallisation, however, is also influenced by insoluble matter and storage temperature [5,62].

Overall, total fructose and glucose contents ranged from 61.71 ± 0.45% to 77.94 ± 0.08%, above the 60 g/100 g threshold of the European Honey Directive [63]. These results are consistent with Algerian (77.72–84.40%) [46], Tunisian (64.71–73.69%) [24], Moroccan (67.06–79.85%) [64], Portuguese (61–78%) [65], and Spanish honeys (63.42–73.43%) [66].

Thus, sugar composition not only confirms the authenticity and quality of Algerian honeys but also highlights variations linked to botanical source, geographical origin, and storage conditions [2].

### 3.9 Chromatic diversity of Algerian honeys

Honey colour is a major quality and classification parameter [27], influenced by floral origin, geographical conditions, and pigment content. Instrumental approaches, particularly the CIELAB colour system, provide a standardised method for assessment [67]. This system describes colour using three coordinates—lightness (L*), red/green (a*), and yellow/blue (b*)—from which chroma ($C_{ab}^{*}$) and hue angle ($h_{a}^{b^{\circ }}$) are derived:


$$C_{ab}^{*}=\;\sqrt{a^{{*}^{\text{2}}}+\;b^{{*}^{\text{2}}}}$$ (2)

$${\text{and }h}_{a}^{b^{\circ }}=\text{arctan}\left(\frac{b^{*}}{a^{*}}\right),$$ (3)

The lightness parameter (L*) ranged from 34.18 ± 0.20 to 60.02 ± 2.47 (Table 4), averaging 44.18. Dark amber honeys such as carob (34.18 ± 0.20, S10), eucalyptus (35.12 ± 2.71, S27), and rosemary (35.19 ± 0.14, S29) exhibited the lowest values, while multifloral (60.02 ± 2.47, S13), harmal (57.84 ± 0.45, S35), and thyme honeys (57.30 ± 0.57, S7) recorded the highest, reflecting their brighter appearance.

**Table 4:** Spectrophotometric analysis of colour data for western Algerian honey samples.

Sample	L*	a*	b*	$C_{ab}^{*}$	$h_{a}^{b^{\circ }}$
S1	37.61 ± 0.76	2.87 ± 0.24	5.31 ± 0.18	6.04 ± 0.24	61.63 ± 1.90
S2	42.80 ± 2.51	2.81 ± 0.21	4.98 ± 0.21	5.72 ± 0.28	60.59 ± 0.85
S3	40.50 ± 0.40	6.79 ± 0.07	5.93 ± 0.05	9.01 ± 0.08	41.15 ± 0.48
S4	40.50 ± 1.12	6.79 ± 0.42	5.93 ± 0.31	9.01 ± 0.52	41.15 ± 0.27
S5	42.74 ± 1.01	2.17 ± 0.02	7.17 ± 0.81	7.49 ± 0.78	73.01 ± 1.81
S6	42.63 ± 0.58	2.23 ± 0.17	11.34 ± 0.83	11.55 ± 0.84	78.85 ± 0.36
S7	57.30 ± 0.57	1.91 ± 0.07	4.81 ± 0.06	5.17 ± 0.08	68.33 ± 0.53
S8	53.69 ± 2.27	1.90 ± 0.29	3.51 ± 0.35	4.01 ± 0.18	61.36 ± 5.98
S9	37.91 ± 0.68	2.89 ± 0.22	4.96 ± 0.30	5.74 ± 0.37	59.79 ± 0.47
S10	34.18 ± 0.20	(−0.57)±0.07	(−0.57)±0.04	0.81 ± 0.06	225.09 ± 3.71
S11	41.51 ± 3.19	1.84 ± 0.37	4.49 ± 0.49	4.85 ± 0.59	67.85 ± 2.06
S12	45.06 ± 1.36	1.13 ± 0.07	5.81 ± 0.24	5.92 ± 0.23	78.93 ± 0.91
S13	60.02 ± 2.47	1.99 ± 0.69	7.94 ± 0.74	8.22 ± 0.55	75.65 ± 5.90
S14	41.14 ± 4.54	0.85 ± 0.23	0.78 ± 0.18	1.18 ± 0.12	43.16 ± 12.35
S15	38.55 ± 0.40	10.21 ± 0.05	7.29 ± 0.37	12.55 ± 0.24	35.50 ± 1.32
S16	38.60 ± 0.40	10.24 ± 0.05	7.30 ± 0.36	12.56 ± 0.17	35.45 ± 1.29
S17	54.43 ± 6.18	2.84 ± 0.35	6.33 ± 0.16	6.94 ± 0.20	65.87 ± 2.75
S18	50.86 ± 0.46	3.02 ± 0.14	13.25 ± 0.86	13.59 ± 0.87	77.17 ± 0.26
S19	42.81 ± 1.76	2.97 ± 0.35	7.77 ± 0.83	8.31 ± 0.90	69.10 ± 0.23
S20	45.34 ± 0.06	2.37 ± 0.04	9.34 ± 0.13	9.64 ± 0.12	75.73 ± 0.45
S21	49.45 ± 2.49	2.32 ± 0.08	5.86 ± 0.44	6.30 ± 0.42	68.31 ± 1.32
S22	48.45 ± 1.78	1.34 ± 0.18	2.32 ± 0.28	2.68 ± 0.33	60.04 ± 0.62
S23	41.79 ± 2.53	8.36 ± 1.40	7.56 ± 1.07	11.27 ± 1.75	42.24 ± 0.87
S24	44.41 ± 2.99	1.10 ± 0.12	5.40 ± 0.40	5.52 ± 0.39	78.41 ± 1.47
S25	37.64 ± 1.13	1.86 ± 0.21	5.56 ± 0.35	5.87 ± 0.40	71.51 ± 0.97
S26	45.97 ± 2.28	2.07 ± 0.09	3.11 ± 0.04	3.74 ± 0.08	56.39 ± 0.79
S27	35.12 ± 2.71	2.35 ± 0.36	3.06 ± 0.07	3.86 ± 0.25	52.71 ± 4.00
S28	41.65 ± 1.10	2.10 ± 0.16	4.53 ± 0.27	4.99 ± 0.31	65.10 ± 0.40
S29	35.19 ± 0.14	2.09 ± 0.04	3.48 ± 0.12	4.06 ± 0.13	59.05 ± 0.44
S30	40.14 ± 0.17	2.01 ± 0.37	6.86 ± 0.12	7.15 ± 0.22	73.68 ± 2.52
S31	37.77 ± 1.70	7.39 ± 0.77	7.80 ± 0.95	10.74 ± 1.22	46.52 ± 0.70
S32	48.36 ± 3.28	2.30 ± 0.16	7.28 ± 0.81	7.64 ± 0.81	72.40 ± 1.18
S33	43.10 ± 0.34	2.74 ± 0.07	8.90 ± 0.32	9.32 ± 0.32	72.88 ± 0.30
S34	49.77 ± 2.60	3.21 ± 0.67	13.84 ± 2.67	14.21 ± 2.75	76.98 ± 0.25
S35	57.84 ± 0.45	1.67 ± 0.11	13.06 ± 0.34	13.17 ± 0.34	82.73 ± 0.38
S36	43.60 ± 0.80	2.41 ± 0.07	13.90 ± 0.38	14.11 ± 0.38	80.18 ± 0.02
S37	46.23 ± 0.35	1.73 ± 0.08	3.37 ± 0.01	3.79 ± 0.04	62.89 ± 0.98
Statistics					
Mean	44.65	3.10	6.28	7.18	66.28
SD	1.68	0.28	0.50	0.51	2.13

All values are reported as the means of triplicate measurements ± standard deviation. L*: clarity (L* = 0, black and L* = 100, colorless); a*: green/red color component (a* > 0, red and a* < 0, green); b*: blue/yellow color component (b* > 0, yellow and b* < 0, blue);${\;C}_{ab}^{*}$: chroma and $h_{a}^{b^{\circ }}$: hue angle. The findings demonstrate statistical significance at **p* *< 0.05.

**Table 5:** Literature-based physicochemical parameters of Polish honeys.

Physical and chemical parameters							
Honey type	Moisture content * (%)	pH *	Free acidity * (meq/kg)	EC * (mS/cm)	HMF * (mg/kg)	Proline * (mg/kg)	${[\alpha ]}_{𝐃}^{20}$ *
multifloral	17.0 [75]	3.87 [76]	30.3 [75]	0.41 [75]	6.91-8.42 [77]	585 [75]	(−11.0)-(−2.2) [78]
	16.9 [76]	4.1 ± 0.2 [79]	30 [76]	0.40 [76]	0.5-13.9 [78]	312.1-443.1 [77]	
	18.6 [80]		11.9-28.7 [78]	0.303-0.584 [81]			
	18.0-20.0 [81]		34.04 ± 25.33 [79]				
	15.7-19.0 [78]						
heather	18.3 [75]	4.07-4.66 [82]	35.7 [75]	0.64 [75]	0.7-14.8 [81]	861 [75]	(−14.35)–(−15.03) [83]
	18.6-19.9 [81]	4.25 ± 0.01 [79]	14.9-33.8 [82]	0.533-0.583 [81]		33.1-92.1 [82]	
	15.4-21.9 [82]	3.65 [76]	32.33 ± 1.03 [79]	0.37-0.82 [82]			
buckwheat	19.9 [75]	3.44-3.80 [54]	54.7 [75]	0.43 [75]	6.4-16.0 [78]	892 [75]	(−12.7)-(−5.3) [78]
	18.5 [76]	4.07 ± 0.16 [79]	45.5 [76]	0.51 [76]	3-79 [54]		(−12.0)-(−7.5) [54]
	16.5 [80]		37.8-50.8 [79]	0.326-0.507 [81]			
	18.1-19.9 [81]		34.25 ± 10.67 [79]				
	16.5-20.8 [78]						
	16.2-20.8 [54]						
**Sugar content.**							
	G * **(%)**	F * **(%)**	(M + S) * **(%)**	(F + G) * **(%)**	F/G *	Total sugar content * (%)	
multifloral	30.22-35.42 [84]	33.72-37.70 [84]	3.50-7.99 [84]	63.94-71.96 [84]	1.03-1.13 [84]	79.5-82.8 [79]	
	34.07-37.74 [77]	41.99-45.24 [77]	1.12-1.27 [77]	56.0-84.1 [77]	1.11-1.32 [77]		
	19.0-36.3 [79]	37.0-52.0 [79]					
heather	30.27-33.55 [84]	37.12-40.92 [84]	4.10-8.42 [84]	67.39-73.94 [84]	1.20-1.27 [84]	71.49-82.36[84]	
	25.9-34.3 [82]	36.5-43.3 [82]	1.3-3.3 [82]	62.4-76.1 [82]	1.12-1.46 [82]	72.0-72.9 [85]	
buckwheat	24.0-31.1 [78]	39.3-53.8 [78]		63.4-80.1 [78]		77.8-82.0 [78]	
						77.6-82.1 [54]	
**Colour data**							
	L*	a*	b*	$C_{ab}^{*}$	$h_{a}^{b^{\circ }}$		
multifloral	57.29 [86]	5.12 [86]	34.90 [86]	22.74 ± 9 [79]	0.05 ± 0.04 [79]		
	40.51 [86]	3.50 [86]	29.94 [86]				
	56.26 [86]	6.56 [86]	37.75 [86]				
	53.7 [80]	1.7 [80]	7.2 [80]				
	42 ± 1.9 [79]	−1.14 ± 1.05 [79]	23.7 ± 8.99 [79]				
heather	26 ± 0.4 [79]	0.54 ± 0.16 [79]	5.8 ± 0.21 [79]	5.83 ± 0.2 [79]	0.09 ± 0.03 [79]		
buckwheat	3.38 [86]	1.89 [86]	3.86 [86]	9.29 ± 3.88 [79]	0.22 ± 0.35 [79]		
	8.40 [86]	8.68 [86]	9.21 [86]				
	12.29 [86]	12.78 [86]	17.7 [86]				
	39.1 [80]	1.8 [80]	−2.6 [80]				
	33 ± 8.7 [79]	2.25 ± 3.84 [79]	8.39 ± 3.48 [79]				

EC: Electrical conductivity; HMF: Hydroxymethyl furfural; ${\left[\alpha \right]}_{\text{D}}^{\text{2}\text{0}}$: Specific optical rotation; G: Glucose; F: Fructose; M: Maltose; S: Sucrose; L*: clarity (L* = 0, black and L* = 100, colorless); a*: green/red color component (a* > 0, red and a* < 0, green); b*: blue/yellow color component (b* > 0, yellow and b* < 0, blue);$C_{ab}^{*}$: chroma and $h_{a}^{b^{\circ }}$: hue angle.

The a* coordinate revealed strong differences. Carob honey (−0.57 ± 0.07, S10) was the only sample with a negative value, showing a slight green hue. In contrast, orange blossom honey (10.24 ± 0.05, S16) exhibited the most intense red tone, followed by multifloral (10.21 ± 0.05, S15), milk thistle (8.36 ± 1.40, S23), and multifloral (7.39 ± 0.77, S31). The lowest positive values were observed in lighter honeys, including S14, S24, S12, and S22 (0.85–1.34).

The b* values, representing the yellow/blue axis, ranged from 0.78 ± 0.18 to 13.90 ± 0.38 (mean = 6.67). Multifloral honey (13.90 ± 0.38, S36) and sage honey (13.84 ± 2.67, S34) were the most yellowish, while darker honeys—euphorbia (2.32 ± 0.28, S22), eucalyptus (3.06 ± 0.07, S27), camphor (3.11 ± 0.04, S26), sweet white mustard (3.37 ± 0.01, S37), rosemary (3.48 ± 0.12, S29), and milk thistle (3.51 ± 0.35, S8)—showed lower values.

Chroma ($C_{ab}^{*}$), which measures colour intensity, varied from 0.81 ± 0.06 (carob, S10) to 14.21 ± 2.75 (sage, S34), with an average of 7.48. High values were recorded in lighter honeys such as sage (S34), multifloral (S36), milk thistle (S18), harmal (S35), orange blossom (S16), and sweet white mustard (S6), whereas darker honeys, notably carob (S10), euphorbia (S22), and camphor (S26), displayed the lowest intensities.

The hue angle ($h_{a}^{b^{\circ }}$) further differentiated samples: orange blossom (S16) and harmal (S35) exhibited the highest values, while dark amber honeys generally showed low values.

Overall, most samples from western Algeria were classified as dark or dark amber. This observation is consistent with their richness in pigments, phenolics, pollen, and minerals, which characterise honeys from subhumid, semiarid, and arid regions. Colour differences across samples of identical floral origin highlight the influence of microclimate. Standardising protocols remains essential, as methodological variations directly affect CIELAB colour coordinates [68].

### 3.10 Comparative quality of Algerian and Polish honeys

#### 3.10.1 Organoleptic and nutraceutical attributes.

Western Algerian honeys present a wide sensory spectrum and notable nutraceutical potential, reflecting diverse floral sources and distinctive terroir. Colour ranges from light yellow to dark brown, a trait commonly associated with elevated polyphenol concentrations and increased antioxidant activity [69,70]. Aroma profiles extend from delicate floral notes to woody or slightly bitter nuances, linked to volatile compounds such as aldehydes and ketones [71]. Texture varies from creamy to crystallised according to moisture and sugar composition (fructose, glucose), contributing to mouthfeel and overall acceptability [2]. Mineral composition further modulates taste, colour and electrical conductivity [42,72,73]. Collectively, high levels of polyphenols, flavonoids and enzymatic activity impart antioxidant, anti-inflammatory and antimicrobial properties, supporting potential nutraceutical uses for metabolic and cardiovascular health [2,70,71,74].

#### 3.10.2 Physicochemical comparability with Polish varietals.

Comparison of Algerian samples (Tables 2–4) with literature values for Polish honeys (Table 5) indicates substantial overlap. Moisture (14.7–20.9%) is comparable to Polish multiflorals (16.9–20.0%), while pH (3.5–5.6) and electrical conductivity (0.16–1.18 mS/cm) fall within Polish nectar-type ranges (≈0.30–0.64 mS/cm). Free acidity (8–40 meq/kg) matches multifloral and heather honeys but remains below typical buckwheat levels (≈55 meq/kg). HMF content averaged ≈15 mg/kg, with only two samples above 40 mg/kg (max ≈ 47 mg/kg), still acceptable under tropical-honey thresholds. Proline concentrations (266–987 mg/kg; mean ≈680 mg/kg) exceed values reported for Polish multiflorals (312–585 mg/kg) yet remain below heather/buckwheat benchmarks (≈860–890 mg/kg), confirming sample maturity. All samples were levorotatory (−14.1 to −8.3°), consistent with reported Polish nectar honeys (−15.0 to −2.2°). Sugar composition (fructose + glucose = 68–81%; F/G ≈ 1.0–1.4) and CIELAB colour metrics (L* 34–60) further indicate authenticity and parity with Polish varietals.

#### 3.10.3 Sensory testing and descriptor analysis.

**Fig 2:**
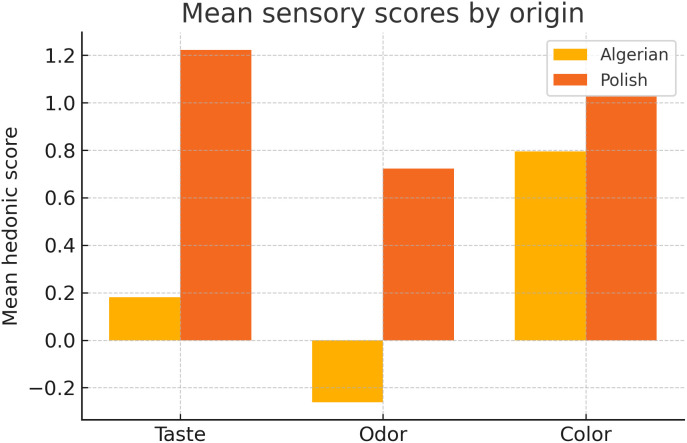
Mean sensory scores by honey origin. p_Taste_ = 0.009, pOdor = 0.016, pColor = 0.459.

Hedonic testing (Fig 2) showed Algerian honeys to be visually comparable to Polish references (colour: p = 0.459) but lower for taste (+0.18 ± 0.52 vs + 1.22 ± 0.42; p = 0.009) and aroma (−0.26 ± 0.36 vs + 0.72 ± 0.43; p = 0.016). Despite intra-group variability, eight Algerian samples (including rosemary S29; multifloral S14 and S31; eucalyptus S5) exceeded the acceptance threshold (> 0.5), qualifying as premium.

**Fig 3:**
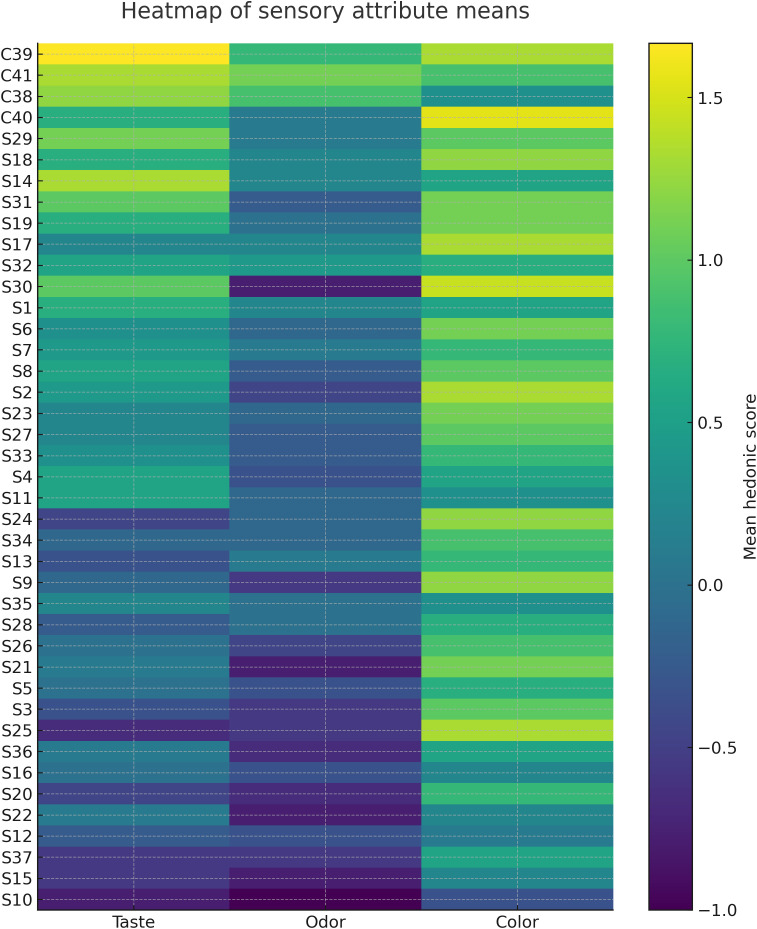
Heat map of the mean sensory attributes for the tested honey samples.

The heat map of mean sensory attributes (Fig 3) provided an overview of perceptual differences between origins, confirming that Algerian samples were generally milder, while Polish honeys displayed stronger sweet and sharp notes.

**Fig 4:**
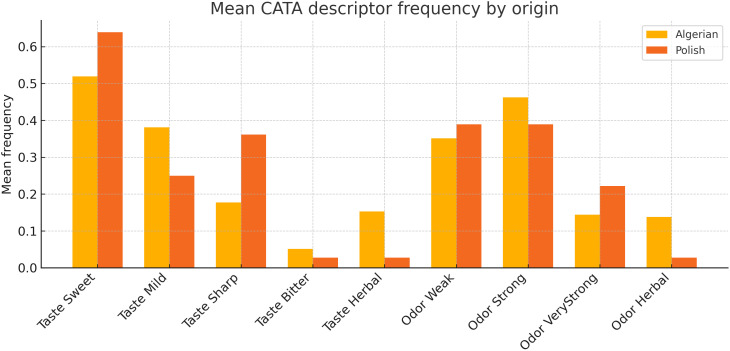
Mean CATA descriptor frequency by honey origin.

**Fig 5:**
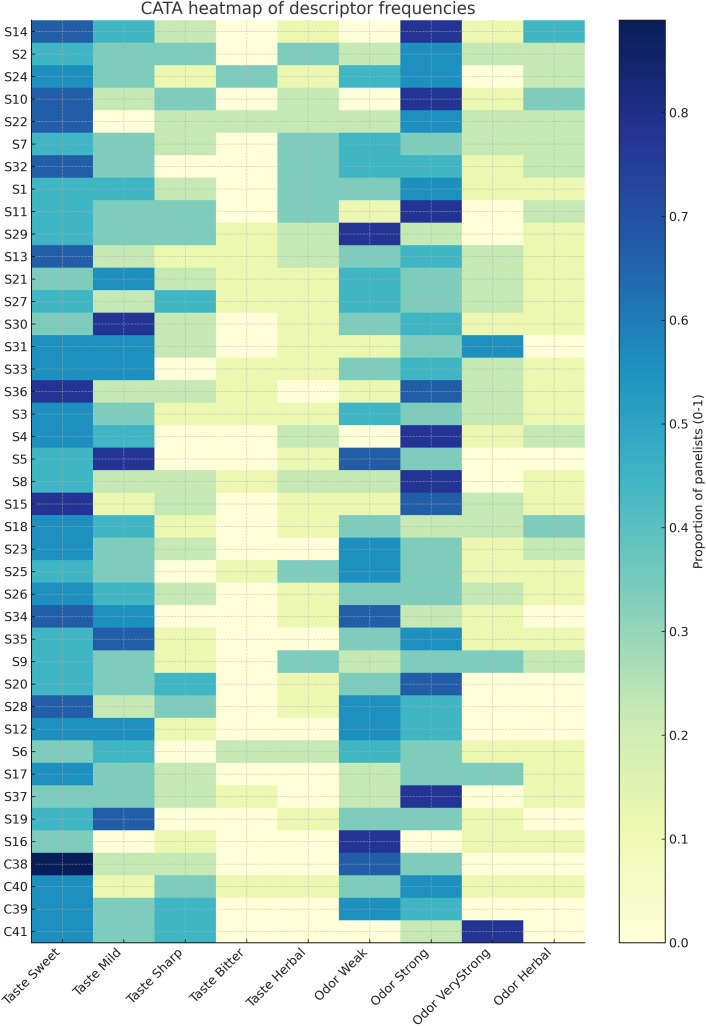
Heat map of CATA descriptor frequencies across honey samples.

The CATA outputs further refined these patterns. Mean descriptor frequencies by honey type (Fig 4) revealed that Algerian samples were more frequently associated with “Taste-Mild” and “Herbal,” while Polish honeys scored higher for “Taste-Sweet” and “Sharp.” The corresponding heat map of descriptor frequencies (Fig 5) highlighted taste as the principal discriminator across samples, whereas colour and aroma intensity were broadly similar between origins.

#### 3.10.4 Multivariate analysis and clustering.

**Fig 6:**
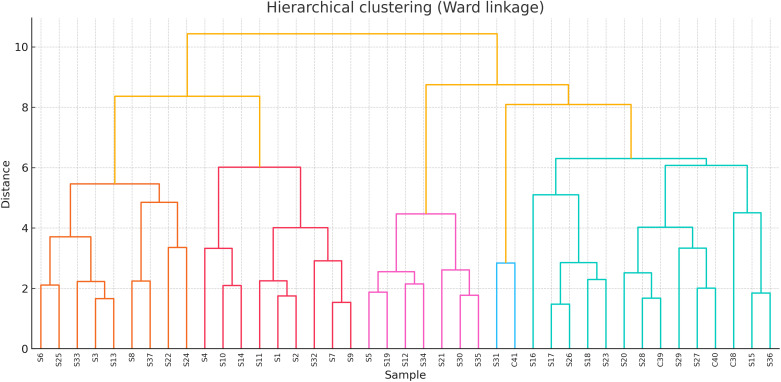
Hierarchical cluster analysis of full CATA sensory profiles for Algerian and Polish honeys.

Hierarchical clustering of the full CATA profile identified three stable clusters: A — Sweet–Aromatic (all four Polish honeys plus four Algerian samples, e.g., S29, S14, S31, S5), B — Mild/Neutral (majority Algerian), and C — Sharp–Bitter/Herbal (six Algerian samples) (Fig 6). The choice of hierarchical clustering (HCA) is scientifically justified by its demonstrated sensitivity in revealing groupings within complex honey sensory and compositional datasets, as exemplified by its use to distinguish volatile compound profiles in honey samples using HS–SPME–GC–MS [87]. This approach was preferred over non-hierarchical methods because HCA preserves nested relationships and yields dendrogram representations that are intuitive for mapping sensory proximities in descriptor-rich data.

**Fig 7:**
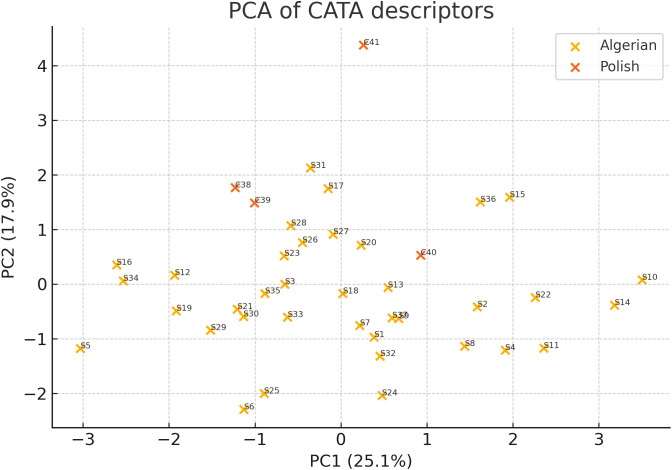
Principal Component Analysis (PC1 vs. PC2) of combined CATA frequencies and hedonic scores (PC1 + PC2 ≈ 65% of variance).

Principal component analysis (PC1 + PC2 ≈ 65% variance; Fig 7) corroborated the cluster structure: PC1 aligned with sweet/strong odour versus sharp/bitter taste, while PC2 related to herbal notes.

#### 3.10.5 Practical implications and market potential.

Descriptor contrasts from CATA (Figs 4 and 5) suggest Algerian honeys are frequently described as “Taste-Mild” and “Herbal,” whereas Polish honeys score as “Taste-Sweet” and “Sharp.” This implies a market niche: positioning selected Algerian varietals as herbal/functional honeys or employing blending strategies to enhance perceived sweetness. Colour parity with Polish products is an advantage for first-impression marketing; taste and aroma limitations can be addressed through targeted varietal selection (e.g., rosemary S29, milk thistle S18) and refined post-harvest handling (gentle heating, controlled creaming).

Given Poland’s import demand (~23,300 t from non-EU sources in 2023) and the limited domestic share of European supply (≈4–6%), there is a tangible commercial opportunity for premium Algerian honeys that meet or exceed quality benchmarks [88,89]. A selective “top-performer” export strategy—promoting honeys that combine high proline, acceptable HMF, and favourable sensory profiles—offers the most viable route to enter premium European niches.

## 4. Conclusions

The integrated physicochemical and sensory evaluation of Western Algerian honeys, in direct comparison with established Polish references, confirmed full compliance with European quality standards. Core compositional indices (moisture, pH, free acidity, HMF, proline, sugar profile, colour) met the required thresholds, while sensory assessment (hedonic scoring, CATA, PCA, hierarchical clustering) revealed three distinct consumer-relevant clusters. Selected samples, notably rosemary (S29) and multifloral honeys (S14, S31), displayed hedonic acceptance and descriptor frequencies comparable to Polish honeys, highlighting their potential for successful positioning in European markets under premium or exotic labels. Certain limitations should nonetheless be acknowledged, particularly the restricted geographic coverage of samples, the moderate number and cultural homogeneity of the sensory panel, and the absence of broader validation across multiple consumer contexts. These factors may constrain the generalisability of the findings. Future research should therefore expand sampling to include additional Algerian regions and harvest seasons, employ larger and more diverse consumer panels within target markets, and integrate longitudinal assessments of product stability. Such developments will strengthen the evidence base for the commercial competitiveness of Algerian honeys in the wider European context.

## Supporting information

S1 File Inclusivity-in-global-research-questionnaire. (DOCX)

S2 File Source data for the results presented in the article. (ZIP)

## References

[1] Kumar D, Hazra K, Prasad PVV, Bulleddu R. Honey: an important nutrient and adjuvant for maintenance of health and management of diseases. J Ethn Food. 2024;11(1):19. doi: 10.1186/s42779-024-00229-3

[2] da Silva PM, Gauche C, Gonzaga LV, Costa ACO, Fett R. Honey: chemical composition, stability and authenticity. Food Chem. 2016;196:309–23. doi: 10.1016/j.foodchem.2015.09.051 26593496

[3] Mwangi MW, Wanjau TW, Omwenga EO. Stingless bee honey: nutritional, physicochemical, phytochemical and antibacterial validation properties against wound bacterial isolates. PLoS One. 2024;19(5):e0301201. doi: 10.1371/journal.pone.0301201 38743750 PMC11093306

[4] Chepulis L. Honey – food or medicine? The how, where, and why of bioactivity testing. Bee World. 2015;92(4):112–5. doi: 10.1080/0005772x.2016.1152847

[5] Akpınar S, Mutlu N. Multidimensional analysis of honey from Eastern Anatolia (Kars): Pollen spectrum, physicochemical properties, and antimicrobial activity. PLoS One. 2025;20(7):e0327861. doi: 10.1371/journal.pone.0327861 40632713 PMC12240352

[6] Solayman M, Islam MA, Paul S, Ali Y, Khalil MI, Alam N, et al. Physicochemical properties, minerals, trace elements, and heavy metals in honey of different origins: a comprehensive review. Compr Rev Food Sci Food Saf. 2016;15(1):219–33. doi: 10.1111/1541-4337.12182 33371579

[7] Thrasyvoulou A, Tananaki C, Goras G, Karazafiris E, Dimou M, Liolios V, et al. Legislation of honey criteria and standards. J Apic Res. 2018;57(1):88–96. doi: 10.1080/00218839.2017.1411181

[8] Codex Committee on Food Hygiene. Report of the thirty-third session of the Codex Committee on Food Hygiene. Rome: Joint FAO/WHO Food Standards Programme, Codex Alimentarius Commission; 2000.

[9] Tamali HS, Özkırım A. Beekeeping activities in Turkey and Algeria. Mellifera. 2019;19:30–40.

[10] Bargańska Ż, Ślebioda M, Namieśnik J. Honey bees and their products: bioindicators of environmental contamination. Crit Rev Environ Sci Technol. 2015;46(3):235–48. doi: 10.1080/10643389.2015.1078220

[11] Schievano E, Finotello C, Uddin J, Mammi S, Piana L. Objective definition of monofloral and polyfloral honeys based on NMR metabolomic profiling. J Agric Food Chem. 2016;64(18):3645–52. doi: 10.1021/acs.jafc.6b00619 27086991

[12] Puścion-Jakubik A, Borawska MH, Socha K. Modern methods for assessing the quality of bee honey and botanical origin identification. Foods. 2020;9(8):1028. doi: 10.3390/foods9081028 32751938 PMC7466300

[13] Adimasu Abeshu M. Medicinal uses of honey. Biol Med. 2015;08(02):276. doi: 10.4172/0974-8369.1000276

[14] Rezazadeh A, Mehrabian AR, Maleki H, Shakoori Z, Golbaghi NZ, Sharifi T, et al. Evaluation of bee pollen by characterizing its botanical origin, total phenolic content, and microbial load for the formulation of apitherapy products. PLoS One. 2025;20(9):e0327480. doi: 10.1371/journal.pone.0327480 40961030 PMC12443248

[15] Bratosin ED, Tit DM, Pasca MB, Purza AL, Bungau G, Marin RC, et al. Physicochemical and sensory evaluation of Romanian monofloral honeys from different supply chains. Foods. 2025;14(13):2372. doi: 10.3390/foods14132372 40647124 PMC12248778

[16] Pattamayutanon P, Angeli S, Thakeow P, Abraham J, Disayathanoowat T, Chantawannakul P. Volatile organic compounds of Thai honeys produced from several floral sources by different honey bee species. PLoS One. 2017;12(2):e0172099. doi: 10.1371/journal.pone.0172099 28192487 PMC5305196

[17] Lopéz-Galán B, de-Magistris T. Exploring consumer preferences and policy implications in local food systems: Does taste or labeling matter in honey? Agric Econ. 2025;13(1):4. doi: 10.1186/s40100-025-00347-9

[18] Manickavasagam G, Saaid M, Lim V. Impact of prolonged storage on quality assessment properties and constituents of honey: a systematic review. J Food Sci. 2024;89(2):811–33. doi: 10.1111/1750-3841.16921 38224177

[19] Bogdanov S. Physical properties of honey. In: Bogdanov S, editor. Book of honey; 2009.

[20] da Costa IF, Toro MJU. Evaluation of the antioxidant capacity of bioactive compounds and determination of proline in honeys from Pará. J Food Sci Technol. 2021;58(5):1900–8. doi: 10.1007/s13197-020-04701-1 33897026 PMC8021653

[21] International Organization for Standardization. ISO 8586:2023: Sensory analysis — selection and training of sensory assessors. Geneva: ISO; 2023.

[22] Moumeh B, Dolores Garrido M, Diaz P, Peñaranda I, Linares MB. Chemical analysis and sensory evaluation of honey produced by honeybee colonies fed with different sugar pastes. Food Sci Nutr. 2020;8(11):5823–31. doi: 10.1002/fsn3.1843 33282234 PMC7684596

[23] Aazza S, Lyoussi B, Antunes D, Miguel MG. Physicochemical characterization and antioxidant activity of 17 commercial Moroccan honeys. Int J Food Sci Nutr. 2014;65(4):449–57. doi: 10.3109/09637486.2013.873888 24438231

[24] Boussaid A, Chouaibi M, Attouchi S, Hamdi S, Ferrari G. Classification of Southern Tunisian honeys based on their physicochemical and textural properties. Int J Food Prop. 2018;21(1):2590–609. doi: 10.1080/10942912.2018.1540988

[25] Akgün N, Çelik ÖF, Kelebekli L. Physicochemical properties, total phenolic content, and antioxidant activity of chestnut, rhododendron, acacia and multifloral honey. Food Meas. 2021;15(4):3501–8. doi: 10.1007/s11694-021-00937-3

[26] Laredj H, Waffa R. Microbiological and physicochemical characterization of honeys from the Tiaret Region of Algeria. AJPRHC. 2017;9(3):85. doi: 10.18311/ajprhc/2017/15698

[27] El Sohaimy SA, Masry SHD, Shehata MG. Physicochemical characteristics of honey from different origins. Ann Agric Sci. 2015;60(2):279–87. doi: 10.1016/j.aoas.2015.10.015

[28] Singh I, Singh S. Honey moisture reduction and its quality. J Food Sci Technol. 2018;55(10):3861–71. doi: 10.1007/s13197-018-3341-5 30228384 PMC6133847

[29] Lewoyehu M, Amare M. Comparative assessment on selected physicochemical parameters and antioxidant and antimicrobial activities of honey samples from selected districts of the Amhara and Tigray Regions, Ethiopia. Int J Food Sci. 2019;2019:4101695. doi: 10.1155/2019/4101695 30949493 PMC6425412

[30] Almasi R, Sekarappa B. Analysis of unifloral and multifloral honey for physico-chemical properties in southern Karnataka, India. Int J Recent Sci Res. 2019;10(5E):32469–73.

[31] Codex Alimentarius Commission. Codex alimentarius: standard for honey (CODEX STAN 12-1981, revised 2001) [Internet]. Rome: FAO/WHO; 2001 [cited 2025 Jul 19]. Available from: https://www.fao.org/fao-who-codexalimentarius

[32] Makhloufi C, Ait Abderrahim L, Taibi K. Characterization of some Algerian honeys belonging to different botanical origins based on their physicochemical properties. Iran J Sci Technol Trans Sci. 2021;45(1):189–99. doi: 10.1007/s40995-020-01047-3

[33] Bella GD, Licata P, Potortì AG, Crupi R, Nava V, Qada B, et al. Mineral content and physico-chemical parameters of honey from North regions of Algeria. Nat Prod Res. 2022;36(2):636–43. doi: 10.1080/14786419.2020.1791110 32643412

[34] Homrani M, Escuredo O, Rodríguez-Flores MS, Fatiha D, Mohammed B, Homrani A, et al. Botanical origin, pollen profile, and physicochemical properties of Algerian honey from different bioclimatic areas. Foods. 2020;9(7):938. doi: 10.3390/foods9070938 32708524 PMC7404483

[35] El-Haskoury R, Kriaa W, Lyoussi B, Makni M. Ceratonia siliqua honeys from Morocco: physicochemical properties, mineral contents, and antioxidant activities. J Food Drug Anal. 2018;26(1):67–73. doi: 10.1016/j.jfda.2016.11.016 29389590 PMC9332668

[36] Abdulkhaliq A, Swaileh KM. Physico-chemical properties of multi-floral honey from the West Bank, Palestine. Int J Food Prop. 2016;20(2):447–54. doi: 10.1080/10942912.2016.1166128

[37] Escuredo O, Rodríguez-Flores MS, Rojo-Martínez S, Seijo MC. Contribution to the chromatic characterization of unifloral honeys from Galicia (NW Spain). Foods. 2019;8(7):233. doi: 10.3390/foods8070233 31261909 PMC6678377

[38] Khalafi R, Goli SAH, Behjatian M. Characterization and classification of several monofloral Iranian honeys based on physicochemical properties and antioxidant activity. Int J Food Prop. 2015;19(5):1065–79. doi: 10.1080/10942912.2015.1055360

[39] Ghorab A, Rodríguez-Flores MS, Nakib R, Escuredo O, Haderbache L, Bekdouche F, et al. Sensorial, melissopalynological and physico-chemical characteristics of honey from Babors Kabylia’s region (Algeria). Foods. 2021;10(2):225. doi: 10.3390/foods10020225 33499111 PMC7912395

[40] Uçurum HÖ, Tepe Ş, Yeşil E, Güney F, Karakuş S, Kolayli S, et al. Characterization of Turkish pine honey according to their geographical origin based on physicochemical parameters and chemometrics. Eur Food Res Technol. 2023;249(5):1317–27. doi: 10.1007/s00217-023-04215-y

[41] Recklies K, Peukert C, Kölling-Speer I, Speer K. Differentiation of honeydew honeys from blossom honeys and according to their botanical origin by electrical conductivity and phenolic and sugar spectra. J Agric Food Chem. 2021;69(4):1329–47. doi: 10.1021/acs.jafc.0c05311 33476168

[42] Bereksi-Reguig D, Bouchentouf S, Allali H, Adamczuk A, Kowalska G, Kowalski R. Trace elements and heavy metal contents in west Algerian natural honey. J Anal Methods Chem. 2022;2022:7890856. doi: 10.1155/2022/7890856 36619658 PMC9822738

[43] Silva LR, Sousa A, Taveira M. Characterization of Portuguese honey from Castelo Branco region according to their pollen spectrum, physicochemical characteristics and mineral contents. J Food Sci Technol. 2017;54(8):2551–61. doi: 10.1007/s13197-017-2700-y 28740313 PMC5502051

[44] Fechner DC, Moresi AL, Ruiz Díaz JD, Pellerano RG, Vazquez FA. Multivariate classification of honeys from Corrientes (Argentina) according to geographical origin based on physicochemical properties. Food Biosci. 2016;15:49–54. doi: 10.1016/j.fbio.2016.05.002

[45] Zerrouk S, Bahloul R. Palynological and physicochemical properties of multifloral honey produced in some regions of Algeria. J Apic Res. 2020;62(2):345–54. doi: 10.1080/00218839.2020.1856559

[46] Gonçalves J, Ribeiro I, Marçalo J, Rijo P, Faustino C, Pinheiro L. Physicochemical, antioxidant and antimicrobial properties of selected Portuguese commercial monofloral honeys. JFNR. 2018;6(10):645–54. doi: 10.12691/jfnr-6-10-5

[47] Mouhoubi-Tafinine Z, Ouchemoukh S, Bachir Bey M, Louaileche H, Tamendjari A. Effect of storage on hydroxymethylfurfural and color of some Algerian honey. Int Food Res J. 2018;25:1044–50.

[48] Guerzou M, Aouissi HA, Guerzou A, Burlakovs J, Doumandji S, Krauklis AE. From the beehives: identification and comparison of physicochemical properties of Algerian honey. Resources. 2021;10(10):94. doi: 10.3390/resources10100094

[49] Imtara H, Elamine Y, Lyoussi B. Physicochemical characterization and antioxidant activity of Palestinian honey samples. Food Sci Nutr. 2018;6(8):2056–65. doi: 10.1002/fsn3.754 30510707 PMC6261158

[50] Bouhlali E dine T, Bammou M, Sellam K, El Midaoui A, Bourkhis B, Ennassir J, et al. Physicochemical properties of eleven monofloral honey samples produced in Morocco. Arab J Basic Appl Sci. 2019;26(1):476–87. doi: 10.1080/25765299.2019.1687119

[51] Bentabol Manzanares A, Hernández García Z, Rodríguez Galdón B, Rodríguez Rodríguez E, Díaz Romero C. Physicochemical characteristics of minor monofloral honeys from Tenerife, Spain. LWT Food Sci Technol. 2014;55(2):572–8. doi: 10.1016/j.lwt.2013.09.024 28317747

[52] Issaad FZ, Bouhedjar K, Ikhlef A, Lachlah H, Smain DH, Boutaghane K, et al. Multivariate analysis of physico-chemical, bioactive, microbial and spectral data characterisation of Algerian honey. Food Meas. 2021;15(4):3634–48. doi: 10.1007/s11694-021-00946-2

[53] Amessis-Ouchemoukh N, Maouche N, Otmani A, Terrab A, Madani K, Ouchemoukh S. Evaluation of Algerian’s honey in terms of quality and authenticity based on the melissopalynology and physicochemical analysis and their antioxidant powers. MNM. 2021;14(3):305–24. doi: 10.3233/mnm-210561

[54] Dżugan M, Grabek-Lejko D, Swacha S, Tomczyk M, Bednarska S, Kapusta I. Physicochemical quality parameters, antibacterial properties and cellular antioxidant activity of Polish buckwheat honey. Food Biosci. 2020;34:100538. doi: 10.1016/j.fbio.2020.100538

[55] Serrano S, Rodríguez I, Moreno R, Rincón F. Detection of key factors affecting specific optical rotation determination in honey. CyTA J Food. 2019;17(1):574–80. doi: 10.1080/19476337.2019.1620338

[56] Aljohar HI, Maher HM, Albaqami J, Al-Mehaizie M, Orfali R, Orfali R, et al. Physical and chemical screening of honey samples available in the Saudi market: an important aspect in the authentication process and quality assessment. Saudi Pharm J. 2018;26(7):932–42. doi: 10.1016/j.jsps.2018.04.013 30416348 PMC6218329

[57] Polatidou K, Nouska C, Tananaki C, Biliaderis CG, Lazaridou A. Physicochemical and rheological characteristics of monofloral honeys-kinetics of creaming-crystallization. Foods. 2025;14(10):1835. doi: 10.3390/foods14101835 40428613 PMC12111295

[58] Devillers J, Morlot M, Pham-Delègue MH, Doré JC. Classification of monofloral honeys based on their quality control data. Food Chem. 2004;86(2):305–12. doi: 10.1016/j.foodchem.2003.09.029

[59] Tappi S, Glicerina V, Ragni L, Dettori A, Romani S, Rocculi P. Physical and structural properties of honey crystallized by static and dynamic processes. J Food Eng. 2021;292:110316. doi: 10.1016/j.jfoodeng.2020.110316

[60] Augustin LSA, Kendall CWC, Jenkins DJA, Willett WC, Astrup A, Barclay AW, et al. Glycemic index, glycemic load and glycemic response: an International Scientific Consensus Summit from the International Carbohydrate Quality Consortium (ICQC). Nutr Metab Cardiovasc Dis. 2015;25(9):795–815. doi: 10.1016/j.numecd.2015.05.005 26160327

[61] Romero CA, Sosa N, Vallejos OA, Navarro AS, Yamul DK, Baldi Coronel BM. Physicochemical, microbiological, and sensory properties of stingless bee honey from Argentina. J Apic Res. 2024;64(3):932–43. doi: 10.1080/00218839.2024.2350310

[62] Piepiórka-Stepuk J, Sterczyńska M, Stachnik M, Pawłowski P. Effects of refrigerated storage on the physicochemical, color and rheological properties of selected honey. Agriculture. 2025;15(14):1476. doi: 10.3390/agriculture15141476

[63] Inaudi P, Garzino M, Abollino O, Malandrino M, Giacomino A. Honey: inorganic composition as possible marker for botanical and geological assignment. Molecules. 2025;30(7):1466. doi: 10.3390/molecules30071466 40286042 PMC11990508

[64] Bouddine T, Laaroussi H, Bakour M, Guirrou I, Khallouki F, Mazouz H, et al. Organic honey from the middle atlas of Morocco: physicochemical parameters, antioxidant properties, pollen spectra, and sugar profiles. Foods. 2022;11(21):3362. doi: 10.3390/foods11213362 36359976 PMC9658496

[65] Machado AM, Tomás A, Russo-Almeida P, Duarte A, Antunes M, Vilas-Boas M, et al. Quality assessment of Portuguese monofloral honeys. Physicochemical parameters as tools in botanical source differentiation. Food Res Int. 2022;157:111362. doi: 10.1016/j.foodres.2022.111362 35761624

[66] Jara-Palacios MJ, Ávila FJ, Escudero-Gilete ML, Gómez Pajuelo A, Heredia FJ, Hernanz D, et al. Physicochemical properties, colour, chemical composition, and antioxidant activity of Spanish Quercus honeydew honeys. Eur Food Res Technol. 2019;245(9):2017–26. doi: 10.1007/s00217-019-03316-x

[67] Bayram NE, Kara HH, Can AM, Bozkurt F, Akman PK, Vardar SU, et al. Characterization of physicochemical and antioxidant properties of Bayburt honey from the North-east part of Turkey. J Apic Res. 2020;60(1):46–56. doi: 10.1080/00218839.2020.1812806

[68] Carvalho MJ, Pereira V, Pereira AC, Pinto JL, Marques JC. Evaluation of wine colour under accelerated and oak-cask ageing using CIELab and chemometric approaches. Food Bioprocess Technol. 2015;8(11):2309–18. doi: 10.1007/s11947-015-1585-x

[69] Beretta G, Granata P, Ferrero M, Orioli M, Maffei Facino R. Standardization of antioxidant properties of honey by a combination of spectrophotometric/fluorimetric assays and chemometrics. Anal Chim Acta. 2005;533(2):185–91. doi: 10.1016/j.aca.2004.11.010

[70] Bereksi-Reguig D, Allali H, Taib N, Aissaoui N, Wlodarczyk-Stasiak M, Kowalski R. Bioactive compounds, antioxidant properties, and antimicrobial profiling of a range of west Algerian honeys: in vitro comparative screening prior to therapeutic purpose. Foods. 2024;13(24):4120. doi: 10.3390/foods13244120 39767065 PMC11675739

[71] Alvarez-Suarez JM, Tulipani S, Romandini S, Bertoli E, Battino M. Contribution of honey in nutrition and human health: a review. Mediterr J Nutr Metab. 2009;3(1):15–23. doi: 10.1007/s12349-009-0051-6

[72] Alves A, Ramos A, Gonçalves MM, Bernardo M, Mendes B. Antioxidant activity, quality parameters and mineral content of Portuguese monofloral honeys. J Food Compos Anal. 2013;30(2):130–8. doi: 10.1016/j.jfca.2013.02.009

[73] Bereksi-Reguig D, Allali H, Bouchentouf S, Adamczuk A, Kowalska G, Kowalski R. Analysis of trace elements and toxic heavy metals in honeys from Tlemcen Province, north-western Algeria. Agric Conspec Sci. 2020;85:367–74.

[74] Alvarez-Suarez JM, Giampieri F, Battino M. Honey as a source of dietary antioxidants: structures, bioavailability and evidence of protective effects against human chronic diseases. Curr Med Chem. 2013;20(5):621–38. doi: 10.2174/092986713804999358 23298140

[75] Majewska E, Drużyńska B, Derewiaka D, Ciecierska M, Wołosiak R. Fizykochemiczne wyróżniki jakości wybranych miodów nektarowych. Bromat Chem Toksykol. 2015;48:440–4.

[76] Kędzierska-Matysek M, Teter A, Daszkiewicz T, Topyła B, Skałecki P, Domaradzki P, et al. Effect of temperature of two-year storage of varietal honeys on 5-hydroxymethylfurfural content, diastase number, and CIE color coordinates. Agriculture. 2025;15(6):652. doi: 10.3390/agriculture15060652

[77] Pacholczyk-Sienicka B, Ciepielowski G, Modranka J, Bartosik T, Albrecht Ł. Classification of Polish natural bee honeys based on their chemical composition. Molecules. 2022;27(15):4844. doi: 10.3390/molecules27154844 35956789 PMC9369904

[78] Zielińska S, Wesołowska M, Bilek M, Dżugan M. The saccharide profile of Polish honeys depending on their botanical origin. J Microbiol Biotechnol Food Sci. 2014;3:387–90.

[79] Kaczmarek A, Muzolf-Panek M, Tomaszewska-Gras J, Konieczny P. Predicting the botanical origin of honeys with chemometric analysis according to their antioxidant and physicochemical properties. Pol J Food Nutr Sci. 2019;69(2):191–201. doi: 10.31883/pjfns/108526

[80] Majewska E, Drużyńska B, Derewiaka D, Ciecierska M, Pakosz P. Comparison of antioxidant properties and color of selected polish honeys and manuka honey. Foods. 2024;13(17):2666. doi: 10.3390/foods13172666 39272431 PMC11394168

[81] Puścion-Jakubik A, Karpińska E, Moskwa J, Socha K. Content of phenolic acids as a marker of polish honey varieties and relationship with selected honey-quality-influencing variables. Antioxidants (Basel). 2022;11(7):1312. doi: 10.3390/antiox11071312 35883803 PMC9312267

[82] Waś E, Rybak-Chmielewska H, Szcześna T, Kachaniuk K, Teper D. Characteristics of Polish unifloral honeys. III. Heather honey (*Calluna vulgaris* L.). J Apic Sci. 2011;55:129–36.

[83] Crane E, Walker P. Honey sources satellite 4. Physical properties, flavour and aroma of some honeys. London: Bee Research Association; 1986.

[84] Krauze A. Sugar spectrum of Polish nectar and honeydew honeys. Acta Aliment Pol. 1999;17:109–17.

[85] Borowska M, Arciuch L, Puścion-Jakubik A, Lewoc D. Zawartość cukrów (fruktozy, glukozy, sacharozy) i proliny w różnych odmianach naturalnych miodów pszczelich. Probl Hig Epidemiol. 2015;96:816–20.

[86] Starowicz M, Ostaszyk A, Zieliński H. The relationship between the browning index, total phenolics, color, and antioxidant activity of Polish-originated honey samples. Foods. 2021;10(5):967. doi: 10.3390/foods10050967 33925034 PMC8146375

[87] Duru ME, Taş M, Çayan F, Küçükaydın S, Tel-Çayan G. Characterization of volatile compounds of Turkish pine honeys from different regions and classification with chemometric studies. Eur Food Res Technol. 2021;247(10):2533–44. doi: 10.1007/s00217-021-03817-8

[88] Eurostat. EU imported €359.3 million worth of honey in 2023 [Internet]. Brussels: Eurostat; 2024 [cited 2025 Jul 19]. Available from: https://ec.europa.eu/eurostat/web/products-eurostat-news/w/ddn-20240520-1

[89] Pawłowska-Tyszko J, Jarka S, Olech I. Food self-sufficiency in the honey market in Poland. Sustainability. 2024;16(21):9373. doi: 10.3390/su16219373

