# Supplementary material for: Comparative physicochemical characterization and sensory profiling of Western Algerian and Polish honeys
Source: PLoS One. 2025 Oct 17;20(10):e0334514. doi: 10.1371/journal.pone.0334514 (PMC12533912; doi:10.1371/journal.pone.0334514)
Supplement: S2 File — (ZIP) [file pone.0334514.s002.zip › Table S1_Supplementary.docx]

**Table S1_Supplementary Information. Geographical origins of honey samples from western regions in Algeria.**

| **Region** | **Sample** | **Flower type** | **Scientific name** | **Botanical family** | **Location** | **GPS coordinates** | **Climate** | **Altitude (m)** | **Harvest season/year** |
| --- | --- | --- | --- | --- | --- | --- | --- | --- | --- |
| Tlemcen | S1 | Lavender | *Lavandula vera* D.C. | [Lamiaceae](https://fr.wikipedia.org/wiki/Lamiaceae) | Sidi Djillali | 34° 28' 00'' N 1° 34' 60'' W | Subhumid | 1470 | Summer 2018 |
|  | S2 | Rosemary | *Rosmarinus officinalis* L. | [Lamiaceae](https://fr.wikipedia.org/wiki/Lamiaceae) | Sidi Djillali | 34° 28' 00'' N 1° 34' 60'' W | Subhumid | 1470 | Spring 2018 |
|  | S3 | Multifloral | Multifloral | - | Sidi Djillali | 34° 28' 00'' N 1° 34' 60'' W | Subhumid | 1470 | Spring 2018 |
|  | S4 | Multifloral | Multifloral | - | Sidi Djillali | 34° 28' 00'' N 1° 35' 00'' W | Subhumid | 1425 | Summer 2017 |
|  | S5 | Multifloral | Multifloral | - | El Aricha | 34° 13' 22" N 1° 15' 21" W | Subhumid | 1270 | Summer 2017 |
|  | S6 | Sweet white mustard | [*Sinapis alba*](https://en.wikipedia.org/wiki/White_mustard) L. | [Brassicaceae](https://en.wikipedia.org/wiki/Brassicaceae) | Aïn Fezza | 34° 52' 45" N 1° 14' 18" W | Subhumid | 846 | Summer 2017 |
|  | S7 | Thyme | *Thymus vulgaris* L. | [Lamiaceae](https://fr.wikipedia.org/wiki/Lamiaceae) | Beni Snous | 34° 38' 35'' N 1° 33' 41'' W | Subhumid | 835 | Spring 2018 |
|  | S8 | Milk thistle | *Silybum marianum* (L.) Gaertn. | [Asteraceae](https://fr.wikipedia.org/wiki/Asteraceae) | Beni Snous | 34° 38' 35'' N 1° 33' 41'' W | Subhumid | 835 | Summer 2018 |
|  | S9 | Multifloral | Multifloral | - | Oued Chouly | 34° 56' 52'' N 1° 03' 17'' W | Subhumid | 705 | Autumn 2017 |
|  | S10 | Carob | *Ceratonia siliqua* L. | [Fabaceae](https://fr.wikipedia.org/wiki/Fabaceae) | Oued Chouly | 34° 56' 52'' N 1° 03' 17'' N | Subhumid | 705 | Autumn 2017 |
|  | S11 | Thyme | *Thymus vulgaris* L. | [Lamiaceae](https://fr.wikipedia.org/wiki/Lamiaceae) | Beni Mester | 34° 52' 00'' N 1° 25' 00'' W | Subhumid | 697 | Spring 2017 |
|  | S12 | Carob | *Ceratonia siliqua* L. | [Fabaceae](https://fr.wikipedia.org/wiki/Fabaceae) | Béni Ghazli | 34° 52' 34'' N 1° 07' 56'' W | Subhumid | 624 | Spring 2017 |
|  | S13 | Multifloral | Multifloral | - | Oued es Safsâf | 34° 55' 60'' N 1° 18' 00" W | Subhumid | 551 | Summer 2018 |
|  | S14 | Multifloral | Multifloral | - | Sebaa Chioukh | 35° 09' 50'' N 1° 21' 27'' W | Subhumid | 514 | Spring 2017 |
|  | S15 | Multifloral | Multifloral | - | Hennaya | 34° 57' 00'' N 1° 22' 00'' W | Subhumid | 429 | Summer 2017 |
|  | S16 | Orange | *Citrus sinensis* [L.](https://fr.wikipedia.org/wiki/Carl_von_Linn%C3%A9) | [Rutaceae](https://fr.wikipedia.org/wiki/Rutaceae) | Remchi | 35° 03' 00'' N 1° 26' 00'' W | Subhumid | 213 | Spring 2017 |
|  | S17 | Multifloral | Multifloral | - | Honaïne | 35° 10' 35'' N 1° 39' 18'' W | Subhumid | 197 | Spring 2018 |
|  | S18 | Milk thistle | *Silybum marianum* (L.) Gaertn. | [Asteraceae](https://fr.wikipedia.org/wiki/Asteraceae) | Honaïne | 35° 10' 35'' N 1° 39' 18'' W | Subhumid | 197 | Summer 2018 |
| Ain-Temouchent | S19 | Multifloral | Multifloral | - | Oulhaça El Gherarba | 35° 13' 00'' N 1° 31' 00'' W | Subhumid | 232 | Spring 2018 |
|  | S20 | Multifloral | Multifloral | - | Beni Ghanem | 35° 15' 16'' N 1° 25' 38'' W | Subhumid | 220 | Summer 2018 |
|  | S21 | Multifloral | Multifloral | - | Bouzedjar | 35° 34' 28" N 1° 10' 01" W | Subhumid | 104 | Spring 2018 |
| Sidi Bel Abbes | S22 | Euphorbia | *Euphorbia* L. | [Euphorbiaceae](https://fr.wikipedia.org/wiki/Euphorbiaceae) | Ras El Ma | 34° 29' 51'' N 0° 49' 10'' W | Semi-arid | 1105 | Spring 2017 |
|  | S23 | Milk thistle | *Silybum marianum* (L.) Gaertn. | [Asteraceae](https://fr.wikipedia.org/wiki/Asteraceae) | Telagh | 34° 47' 06" N 0° 32' 40" W | Semi-arid | 987 | Spring 2017 |
|  | S24 | Multifloral | Multifloral | - | Lamtâr | 35° 04' 14" N 0° 47' 53" W | Semi-arid | 578 | Spring 2017 |
|  | S25 | Eucalyptus | *Eucalyptus globulus* Labill. | Myrtaceae | Sidi Brahim | 35° 15' 38" N 0° 34' 03" W | Semi-arid | 432 | Spring 2017 |
| Mostaganem | S26 | Camphor | *Cinnamomum camphora* [L.](https://fr.wikipedia.org/wiki/Carl_von_Linn%C3%A9) | [Lauraceae](https://fr.wikipedia.org/wiki/Lauraceae) | Sidi Ali | 36° 06' 17" N 0° 25' 24" E | Semi-arid | 216 | Autumn 2017 |
|  | S27 | Eucalyptus | *Eucalyptus globulus* Labill. | Myrtaceae | Mostaganem | 35° 56' 00" N 0° 05' 00" E | Semi-arid | 104 | Summer 2017 |
|  | S28 | Orange | *Citrus sinensis* [L.](https://fr.wikipedia.org/wiki/Carl_von_Linn%C3%A9) | [Rutaceae](https://fr.wikipedia.org/wiki/Rutaceae) | Bouguirat | 35° 45' 05" N 0° 15' 12" E | Semi-arid | 66 | Spring 2017 |
| Mascara | S29 | Rosemary | *Rosmarinus officinalis* L. | [Lamiaceae](https://fr.wikipedia.org/wiki/Lamiaceae) | Djebel Stamboul | 35° 23' 00" N 0° 09' 00" E | Semi-arid | 932 | Spring 2017 |
| Tiaret | S30 | Multifloral | Multifloral | - | Tiaret | 34° 55' 00" N 1° 34' 60" E | Semi-arid | 1189 | Spring 2018 |
| Naâma | S31 | Multifloral | Multifloral | - | Aïn Sefra | 32° 45' 20" N 0° 35' 09" W | Arid | 1073 | Spring 2017 |
|  | S32 | Jujube | [*Ziziphus lotus*](https://fr.wikipedia.org/wiki/Ziziphus_lotus) L. | [Rhamnaceae](https://fr.wikipedia.org/wiki/Rhamnaceae) | Aïn Sefra | 32° 45' 20" N 0° 35' 09" W | Arid | 1073 | Spring 2017 |
|  | S33 | Jujube | [*Ziziphus lotus*](https://fr.wikipedia.org/wiki/Ziziphus_lotus) L. | [Rhamnaceae](https://fr.wikipedia.org/wiki/Rhamnaceae) | Aïn Ben Khelil | 33° 17' 25" N 0° 45' 51" W | Arid | 1156 | Spring 2017 |
|  | S34 | Sage | *Salvia officinalis* L. | [Lamiaceae](https://fr.wikipedia.org/wiki/Lamiaceae) | Naâma | 33° 17' 25" N 0° 45' 51" W | Arid | 1031 | Spring 2017 |
|  | S35 | Harmal | *Peganum harmala* L. | [Zygophyllaceae](https://fr.wikipedia.org/wiki/Zygophyllac%C3%A9es) | Mecheria | 33° 33' 00" N 0° 17' 00" W | Arid | 891 | Spring 2017 |
| Bechar | S36 | Multifloral | Multifloral | - | Djebel Antar | 31° 56' 34" N 1° 55' 52" W | Arid | 1953 | Winter 2017 |
|  | S37 | Sweet white mustard | [*Sinapis alba*](https://en.wikipedia.org/wiki/White_mustard) L. | [Brassicaceae](https://en.wikipedia.org/wiki/Brassicaceae) | Oued Zouzfana | 32° 04 '01" N 1° 14' 27" W | Arid | 830 | Spring 2017 |
