# Supplementary figures and images for: Comparative physicochemical characterization and sensory profiling of Western Algerian and Polish honeys
Source: PLoS One. 2025 Oct 17;20(10):e0334514. doi: 10.1371/journal.pone.0334514 (PMC12533912; doi:10.1371/journal.pone.0334514)

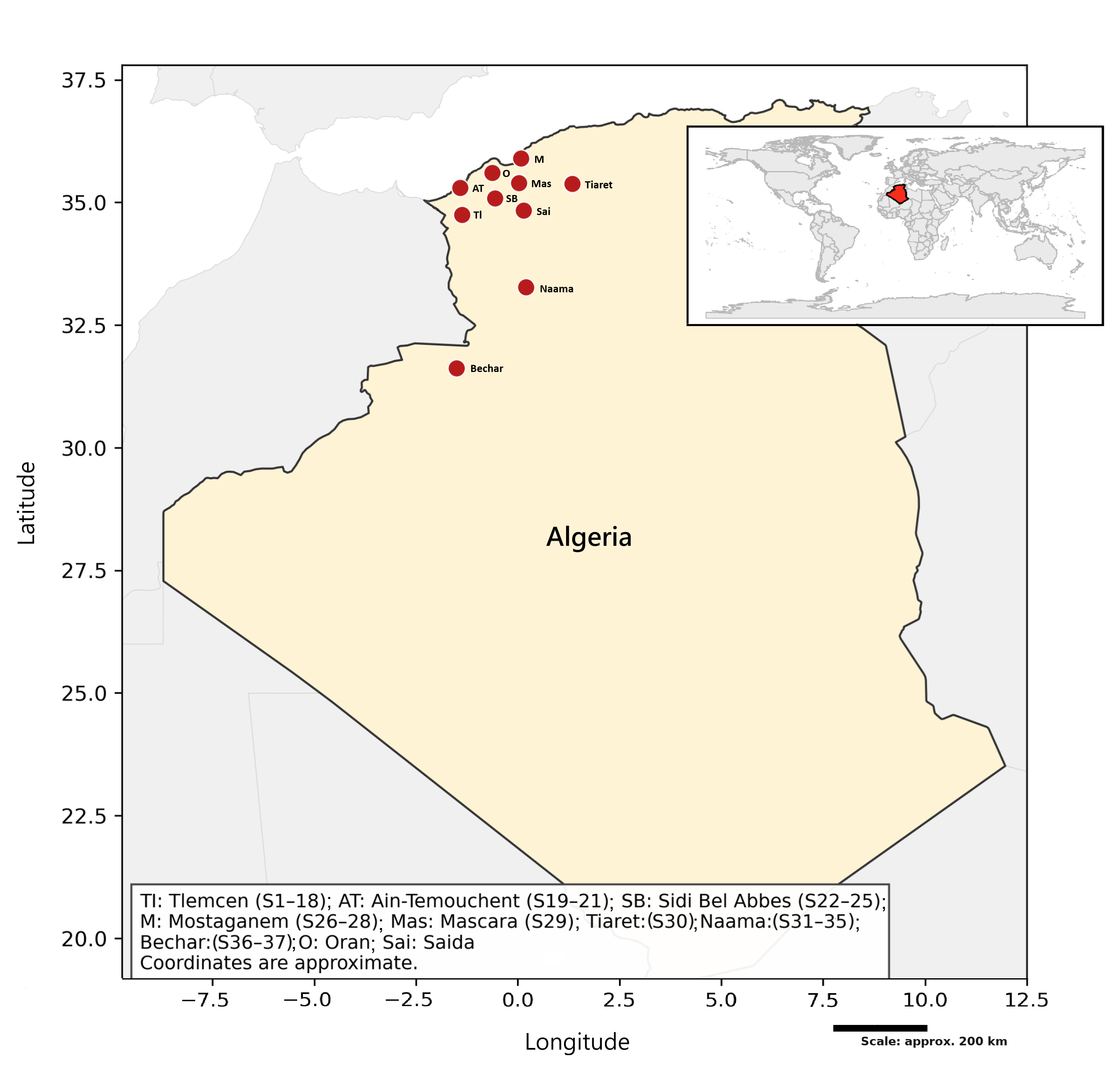

Supplement: S2 File — (ZIP) [file pone.0334514.s002.zip › Fig1.tiff]

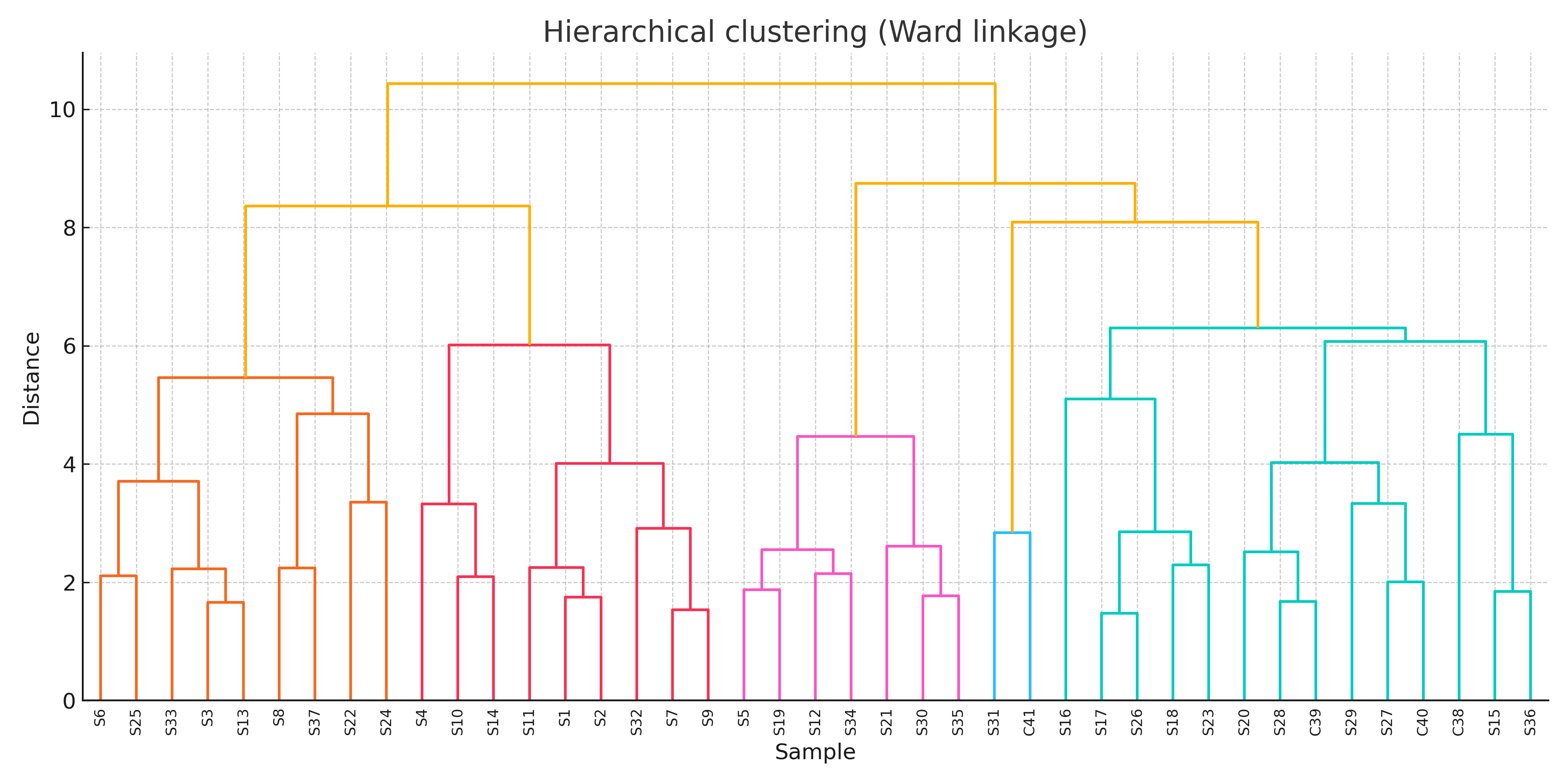

Supplement: S2 File — (ZIP) [file pone.0334514.s002.zip › cata_dendrogram_codes Figure 6.png]

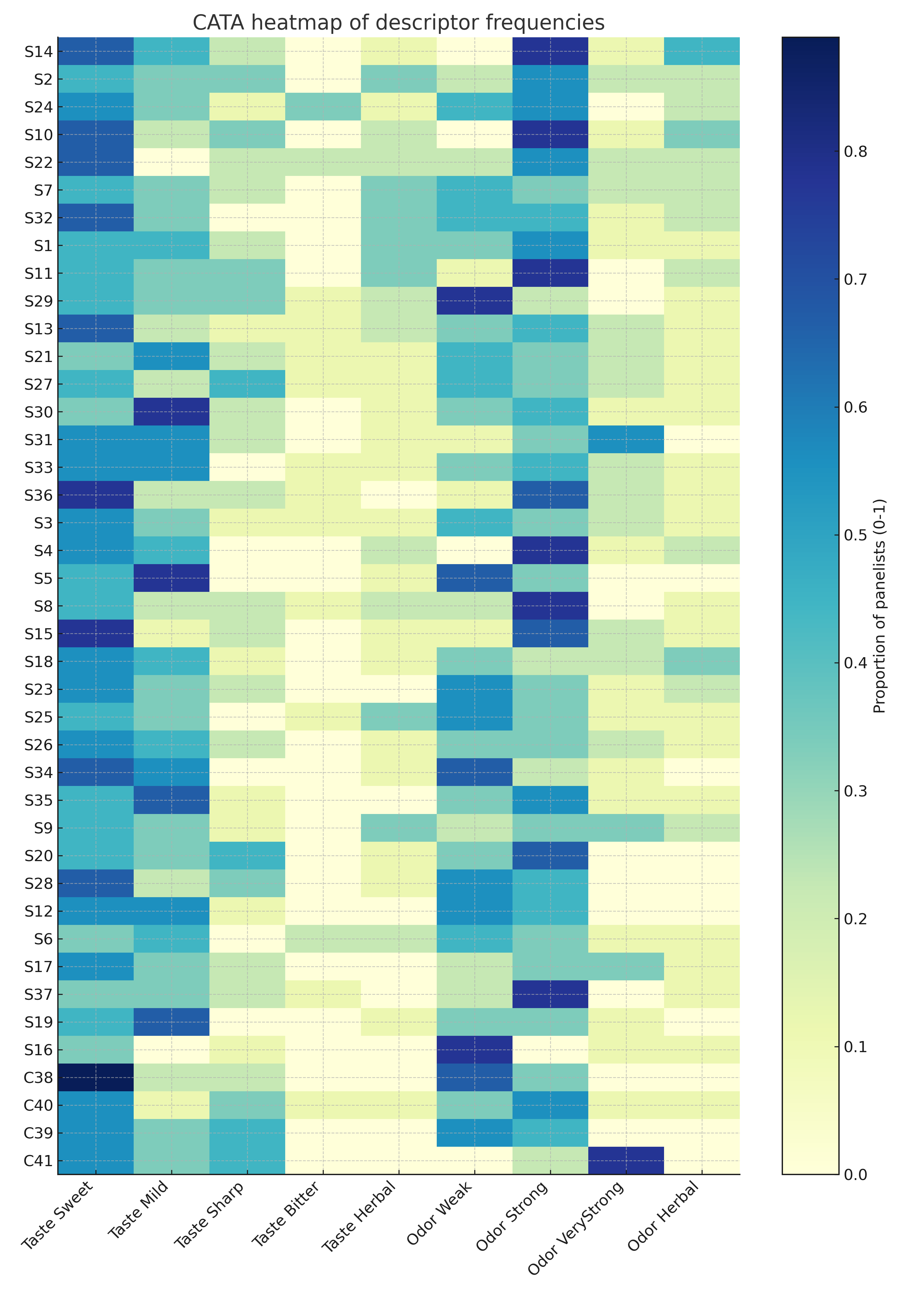

Supplement: S2 File — (ZIP) [file pone.0334514.s002.zip › cata_heatmap_codes Figure 5.png]

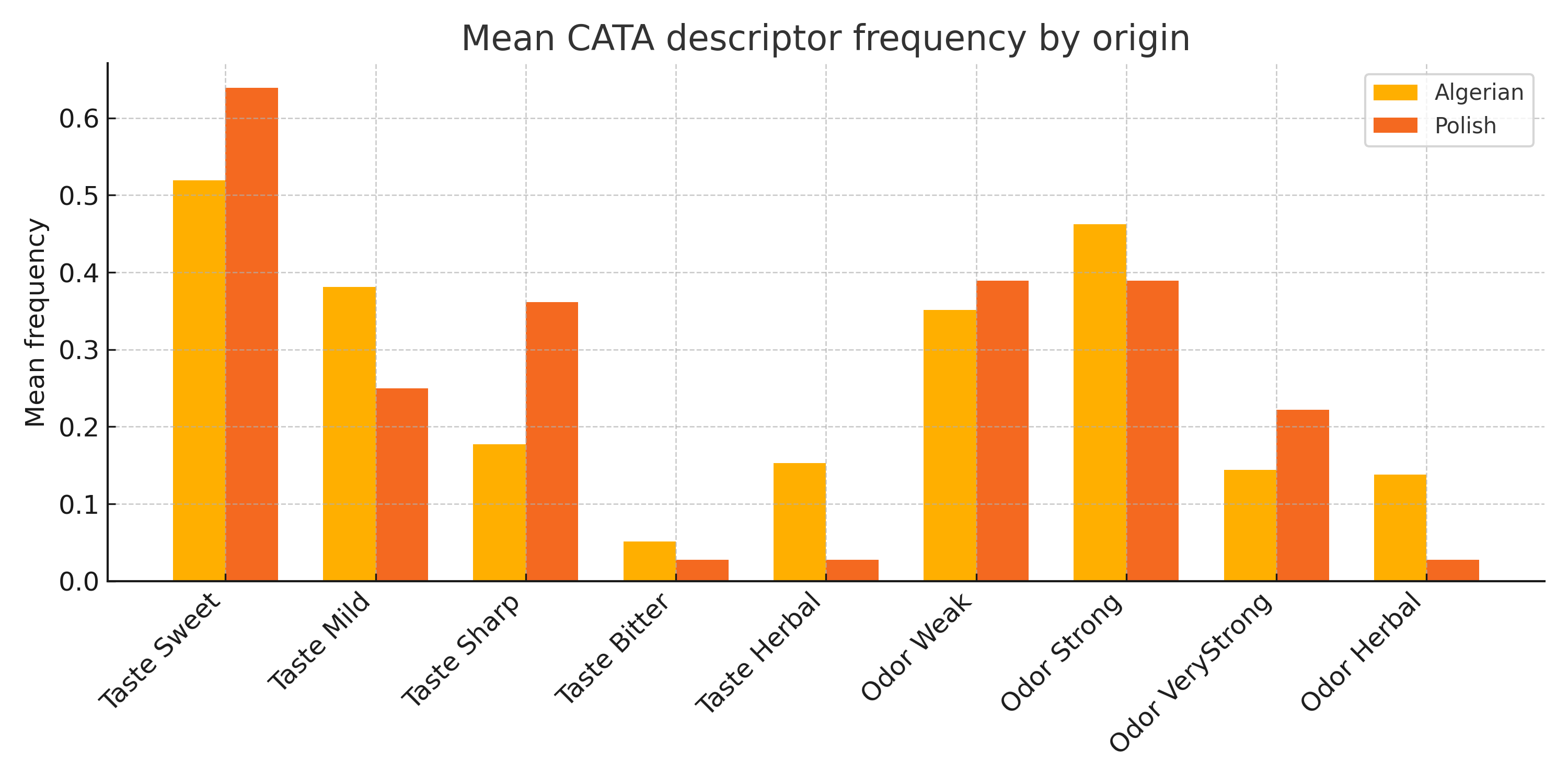

Supplement: S2 File — (ZIP) [file pone.0334514.s002.zip › cata_origin_bar_codes Figure 4.png]

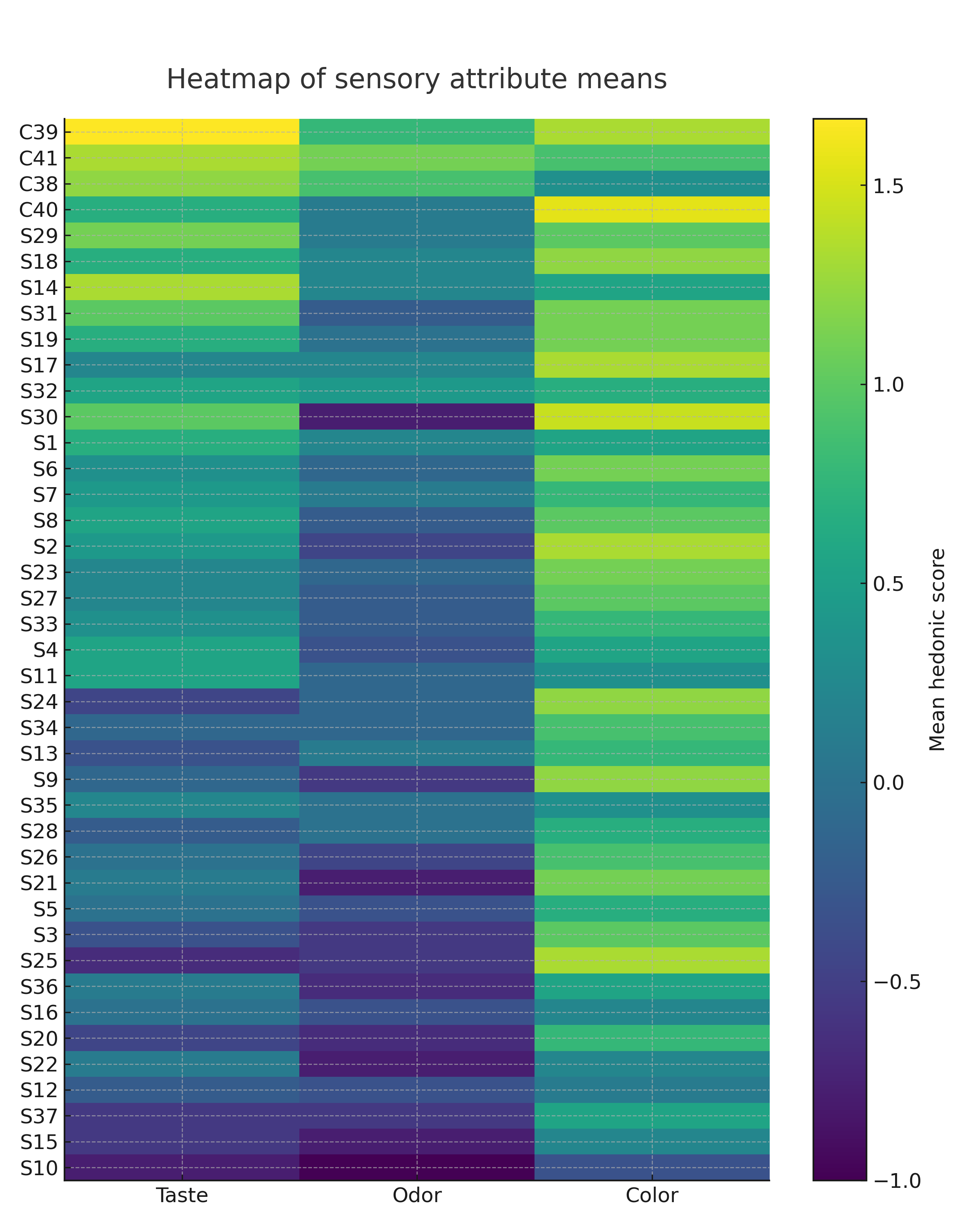

Supplement: S2 File — (ZIP) [file pone.0334514.s002.zip › honey_heatmap_codes_fixed Figure 3.png]

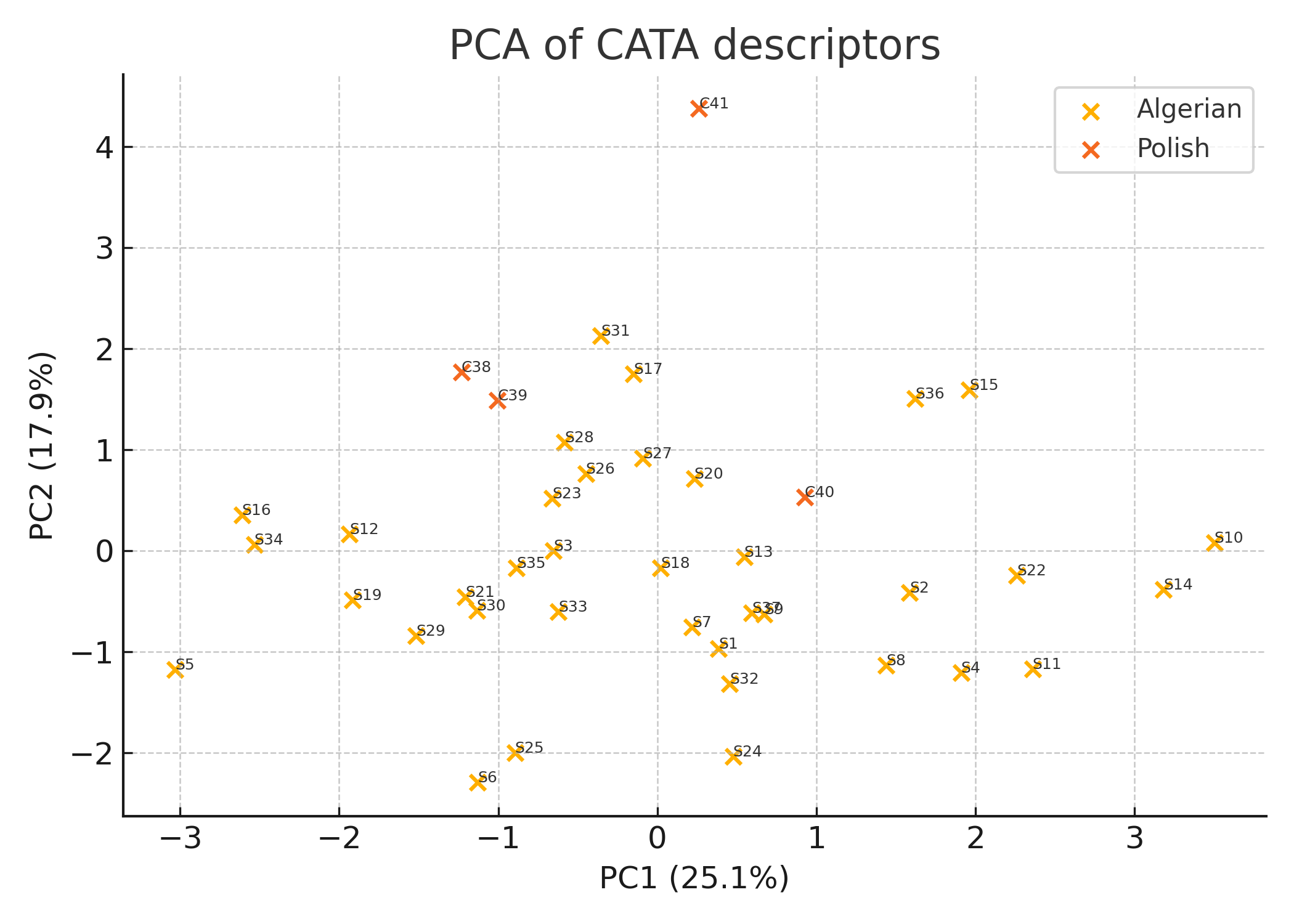

Supplement: S2 File — (ZIP) [file pone.0334514.s002.zip › pca_scatter_codes Figure 7.png]

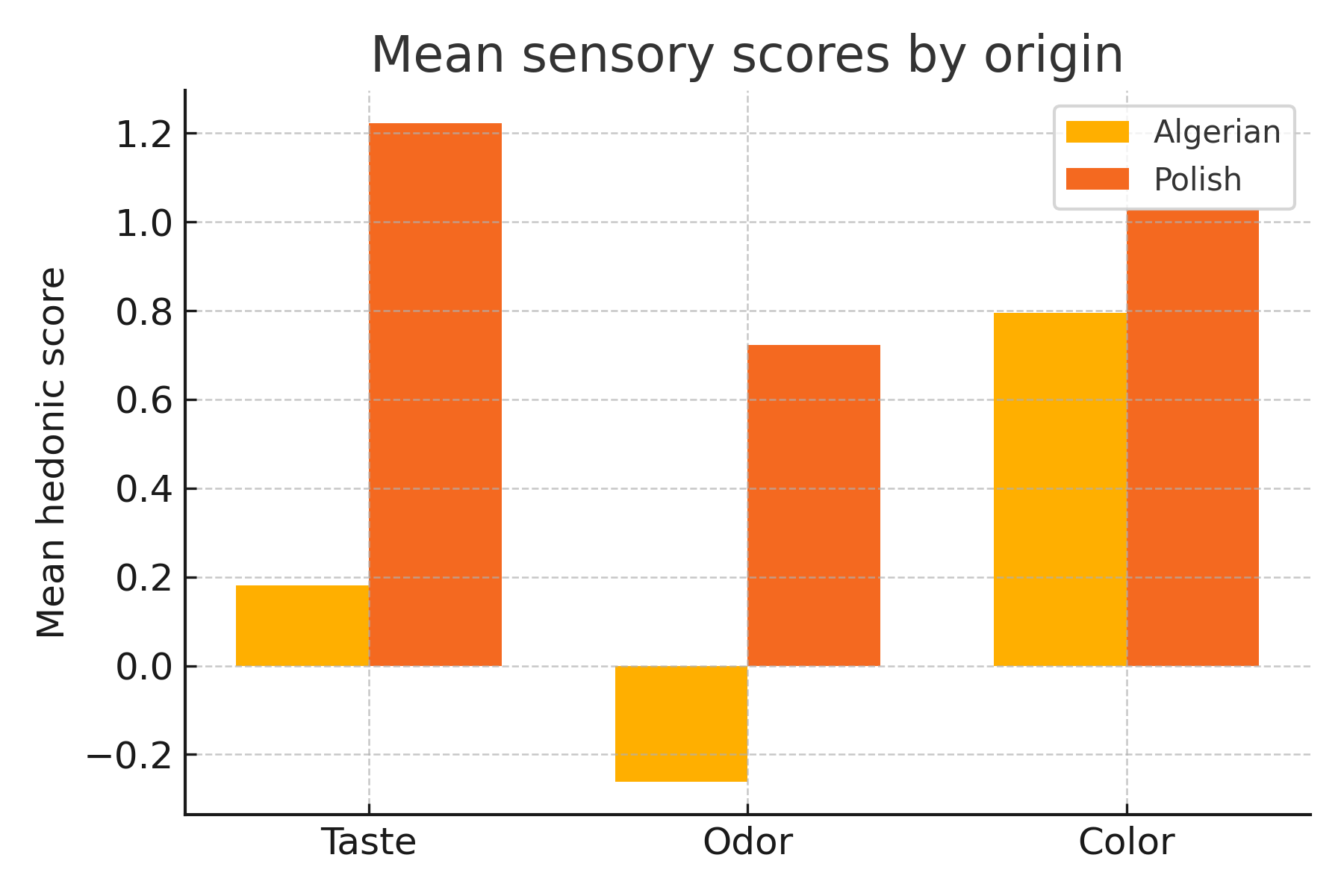

Supplement: S2 File — (ZIP) [file pone.0334514.s002.zip › sensory_origin_bar_updated Figure 2.png]
