# Supplementary material for: Comparative physicochemical characterization and sensory profiling of Western Algerian and Polish honeys
Source: PLoS One. 2025 Oct 17;20(10):e0334514. doi: 10.1371/journal.pone.0334514 (PMC12533912; doi:10.1371/journal.pone.0334514)
Supplement: Attachment — Submitted filename: Response to Reviewers.pdf [file pone.0334514.s004.pdf]

DEPARTMENT OF ANALYSIS
AND EVALUATION OF FOOD QUALITY

UNIVERSITY of LIFE SCIENCES in Lublin

FACULTY OF FOOD SCIENCE AND BIOTECHNOLOGY

Lublin, 08.09.2025

Dear Editor
PLOS One Editorial Office
Manuscript ID: PONE-D-25-40193
Type: Article research
Title: Comparative Physicochemical Characterization and Sensory Profiling of Western Algerian and Polish Honeys
Journal : PLOS ONE

On behalf of all co-authors, I am pleased to resubmit our revised manuscript entitled "Comparative Physicochemical Characterization and Sensory Profiling of Western Algerian and Polish Honeys". (ID: PONE-D-25-40193) to PLOS One. We are grateful for the reviewers' constructive comments, which have helped us improve the manuscript.

Changes to the text are marked in green.

We believe that the revised version fully meets the reviewers' requirements and is now suitable for publication.

Responses to the Reviewers

Reviewer 1:

1. The most significant methodological concern is the 7-year gap between physiochemical analyses (2017-2018) and sensory evaluation (2025). While the authors acknowledge this limitation and cite literature supporting honey stability under refrigerated storage, this temporal separation:

a. May compromise the validity of correlating physicochemical and sensory data.

b. Could introduce confounding variables related to storage-induced changes.

Response to Reviewer 1 – Temporal Gap Between Analyses

We thank the reviewer 1 for highlighting the seven-year gap between the physicochemical analyses (2017–2018) and the sensory evaluation (2025). We fully acknowledge this point. However, several aspects of our study support the validity of our approach. Honey is widely recognised for its stability, owing to its low water activity, high osmotic pressure, and antimicrobial properties, which ensure long-term preservation. Under refrigerated storage at 4 °C, both physicochemical and sensory properties are known to remain stable for extended periods (1. Kędzierska-Matysek, M.; Teter,

A.; Daszkiewicz, T.; Topyła, B.; Skałecki, P.; Domaradzki, P.; Florek, M. Effect of Temperature of Two-Year Storage of Varietal Honeys on 5-Hydroxymethylfurfural Content, Diastase Number, and CIE Color Coordinates. Agriculture 2025, 15, 652. https://doi.org/10.3390/agriculture15060652, 2. Youssef, M. K. E.; El-Rify, M. N. A.; Ramadan, E. A. and Saleh, A. S. M. The Effects of Heating Treatment and Storage Temperature on Some Physico-chemical Properties of Some Egyptian Honey Types after one Year Storage. J. Saudi Soc. for Food and Nutrition., Vol. 1, No. 2; 2006 (https://japksu.com/index.php/SSFN/issue/download/84/24).

In our study, all honey samples were kept consistently refrigerated, and at the time of sensory evaluation, there was no evidence of fermentation, crystallisation, or off-flavours. Additionally, the samples initially complied with the European Directive 2001/110/EC standards, indicating their high baseline quality. The sensory evaluation was designed to assess the genuine organoleptic profiles of the Algerian honeys in comparison with the Polish references, with the observed differences reflecting authentic varietal and geographical signatures.

While this temporal separation does not undermine the scientific validity of our findings, we agree with the reviewer 1 that future studies would benefit from performing both physicochemical and sensory analyses contemporaneously.

Clarification on Sample Storage and Analyses

To address the reviewer's concern regarding the temporal gap between physicochemical and sensory analyses, an additional clarification has been inserted in Section 2.2 (Honey samples) and highlighted in green in the revised manuscript. The new sentence reads as follows:

"*All samples were collected between March 2017 and August 2018 and stored in sealed amber glass containers at 4 °C, protected from light and humidity. Previous studies have shown that honey remains stable under such conditions [17], and the samples exhibited no signs of fermentation, crystallisation, or sensory defects at the time of evaluation in 2025.*"

2. Missing comparative data: No parallel physisochemical analysis of Polish honey samples. The authors relied solely on literature values, preventing direct statistical comparison of compositional parameters between origins. This limitation weakens the comparative aspect of the study and reduces confidence in conclusions about relative quality.

Response to Reviewer 1 – Comparative Physicochemical Data

We thank the reviewer 1 for raising this important point. The four Polish honeys included in our study were used as reference samples for the sensory evaluation and had already

ul. Skromna 8, Lublin 20-704
e-mail: radoslaw.kowalski@up.lublin.pl
https://www.researchgate.net/profile/Radoslaw-Kowalski

been subjected to detailed physicochemical characterisation in previous peer-reviewed publications. For this reason, we did not repeat their physicochemical analyses in our laboratory. Instead, we relied on these published data, which provide robust and well-validated compositional parameters.

This approach ensured that the comparison between Algerian and Polish honeys was grounded in reliable quality benchmarks, while allowing us to focus our experimental efforts on the sensory assessment. By combining established physicochemical data from the literature with new sensory analyses performed directly on the same four Polish honeys, the comparative framework remains both rigorous and scientifically sound.

3. With extensive physicochemical parameter testing, there's potential for Type I error inflation due to multiple comparisons, though this wasn't address with appropriate corrections. Authors might discuss these limitations in detail if possible. Despite these limitations, the statistical approach is generally sound and the results provide valuable insights into Algerian honey characterizes. The temporal gap between analyses is the most significant concern that should be addressed in future research. The multivariate statistical methods are suited for this type of comparative sensory and compositional analysis.

Response to Reviewer 1 – Type I Error and Multiple Comparisons

We appreciate the reviewer's thoughtful observation regarding the potential inflation of Type I error due to multiple comparisons across the physicochemical parameters. It is true that no formal correction (e.g., Bonferroni or false discovery rate) was applied. However, we believe the robustness of our findings is supported by several considerations:

1. High statistical significance: The vast majority of the observed differences were associated with very low p-values (often < 0.001), making it unlikely that they are artefacts of Type I error.
2. Consistency across parameters: The physicochemical results are in close agreement with established ranges reported in the literature for honeys of comparable botanical origins, reinforcing their validity.
3. Multivariate confirmation: Beyond univariate tests, multivariate methods such as principal component analysis (PCA) and hierarchical clustering were employed. These approaches integrate multiple variables simultaneously, providing an internal validation of group differentiation that is less affected by multiple testing issues.

For these reasons, while we acknowledge the theoretical concern of Type I error inflation,

ul. Skromna 8, Lublin 20-704
e-mail: radoslaw.kowalski@up.lublin.pl
https://www.researchgate.net/profile/Radoslaw-Kowalski

we are confident that the conclusions drawn from our analyses remain valid. Future studies with larger datasets could incorporate formal corrections for multiple testing to further strengthen statistical rigor.

Reviewer 2:

The manuscript presents an interesting study; however, the overall length and structure make it somewhat difficult to follow. I provide the following comments and suggestions for improvement:

1. The manuscript is excessively long, which reduces readability and disrupts the flow of the research narrative. Streamlining the content would improve clarity.

Response to Reviewer 2 – Length and Structure of the Manuscript

We sincerely thank the reviewer 2 for this constructive feedback. In response to the comment regarding the manuscript length and structure, we have streamlined the content to improve clarity and ensure a smoother narrative flow. Redundant information was removed, and some sections were condensed to maintain a more focused discussion on the study's core objectives. Additionally, excessive background details have been reduced, making the introduction more concise and directly aligned with the study's aims. These changes are highlighted in green within the revised manuscript for ease of reference.

2. The introduction should explicitly state the significance of the study. The main purpose and rationale for conducting this research need to be clearly articulated.

Response to Reviewer 2 – Significance of the Study

We also appreciate the reviewer's suggestion to explicitly state the significance of the study in the introduction. In response, we have revised the introduction to clarify the main purpose and rationale behind the research. The revised version now succinctly explains why this study is important, outlining the unique contribution of Algerian honeys to the broader understanding of honey composition, sensory profiles, and market potential. As noted by Reviewer 3, this suggestion was also taken into account, and the introduction has been improved to better articulate the significance and objectives of the study.

Both Reviewer 2 and Reviewer 3 have raised valid points regarding the length and clarity of the introduction. We have incorporated their suggestions to improve the manuscript's overall structure and ensure the study's significance is clearly communicated. All modifications, as

UNIVERSITY of LIFE SCIENCES in LUBLIN | Department of Analysis and Evaluation of Food Quality
ul. Skromna 8, Lublin 20-704
e-mail: radoslaw.kowalski@up.lublin.pl
https://www.researchgate.net/profile/Radoslaw-Kowalski

mentioned, are highlighted in green for ease of verification.

3. Section 2 is well organized with clear subsections. Nevertheless, the literature review could be strengthened by incorporating earlier studies and comparing them with more recent findings to highlight the novelty of the present work.

Response to Reviewer 2 – Literature Review

We thank the reviewer for this constructive suggestion. In the revised manuscript, the literature review has been strengthened by integrating earlier foundational studies and contrasting them with more recent findings to better contextualise the novelty of our work. These additions are highlighted in green in Section 2.

4. A flowchart or schematic diagram illustrating the research process from beginning to end would greatly enhance the reader's understanding.

Response to Reviewer 2 – Flowchart Suggestion

We appreciate the reviewer's suggestion regarding the inclusion of a schematic diagram. While we agree that such visual tools can be useful, the current manuscript already contains multiple figures and tables that comprehensively illustrate the workflow, results, and interpretations. To avoid redundancy and further lengthening of the manuscript, we respectfully consider that the existing visual material sufficiently guides the reader through the research process.

5. The Results and Discussion section is overly lengthy. Separating the findings into distinct subsections would improve readability and help the reader follow the discussion more effectively.

Response to Reviewer 2 — Results and Discussion structure

We thank the reviewer for this constructive suggestion. In the revised manuscript, the Results and Discussion section has been restructured for greater clarity and readability. Specifically, we have divided the content into distinct subsections reflecting the main analytical aspects (physicochemical parameters, sugar profile, colour analysis, amino acid content, and sensory evaluation). In addition, redundant passages were shortened, and transitions between parts were improved. All these modifications are highlighted in green in the revised version to facilitate verification.

6. The use of hierarchical clustering in this study is not sufficiently justified. No supporting references are provided to indicate that this is the most appropriate method. A

ul. Skromna 8, Lublin 20-704
e-mail: radoslaw.kowalski@up.lublin.pl
https://www.researchgate.net/profile/Radoslaw-Kowalski

clear rationale should be presented, including why hierarchical clustering was chosen over alternative clustering approaches.

Response to Reviewer 2 — Justification for the use of HCA

We appreciate the reviewer's valuable observation. In the revised manuscript, we have added a clarifying sentence (highlighted in green) to explicitly justify the use of hierarchical clustering analysis (HCA): "*The choice of hierarchical clustering (HCA) is scientifically justified by its demonstrated sensitivity in revealing groupings within complex honey sensory and compositional datasets, as exemplified by its use to distinguish volatile compound profiles in honey samples using HS–SPME–GC–MS (e.g. Duru et al., 2021). This method was preferred over non-hierarchical clustering given its capacity to display nested relationships among sensory signatures in our dataset.*"

HCA was chosen because it requires no a priori assumption on the number of clusters, allowing natural sensory groupings to emerge. Its dendrogram structure preserves nested relationships, enabling interpretation at both broad and fine levels. Unlike partitioning methods (e.g., k-means), HCA accommodates irregular cluster shapes common in sensory data. Its results were cross-validated with PCA (PC1 + PC2 ≈ 65% variance), confirming the robustness of the groupings. This approach aligns with previous honey studies combining sensory or volatile data with PCA and HCA (Duru et al., 2021).

Furthermore, the supporting reference (Duru et al., 2021) has been added to the bibliography to substantiate this justification. We hope this clarification addresses the reviewer's concern; additional methodological details can be provided if requested.

We note that recent methodological papers (e.g. Gere 2023: *Attila Gere, Recommendations for validating hierarchical clustering in consumer sensory projects, Current Research in Food Science, Volume 6,2023,100522,ISSN 2665-9271,https://doi.org/10.1016/j.crfs.2023.100522*; Kumar & Chambers 2024: *Kumar R and Chambers E IV (2024) Unreliability of clustering results in sensory studies and a strategy to address the issue. Front. Food. Sci. Technol. 4:1271193. doi: 10.3389/frfst.2024.1271193*) have discussed the strengths and potential limitations of HCA in sensory research. While these reinforce the importance of validation, we consider HCA appropriate for our dataset, as it has been successfully applied in honey classification (Duru et al. 2021) and produced stable clusters corroborated by PCA.

7. The conclusion section should be expanded to include a more detailed discussion of

ul. Skromna 8, Lublin 20-704
e-mail: radoslaw.kowalski@up.lublin.pl
https://www.researchgate.net/profile/Radoslaw-Kowalski

the study's limitations as well as recommendations for future research.

Response to Reviewer 2 – Conclusion

We thank the reviewer for this valuable suggestion. In the revised version, the conclusion has been expanded to incorporate a balanced discussion of both limitations and future directions. The limitations now acknowledge the temporal separation between physicochemical and sensory analyses as well as the restricted regional scope of sampling. In terms of recommendations, the conclusion emphasises the importance of extending sampling to other Algerian regions, conducting contemporaneous physicochemical and sensory assessments, and including broader consumer panels in target markets to strengthen external validity. These additions, highlighted in green in the manuscript, provide a more complete and forward-looking conclusion in line with the reviewer's request.

Reviewer 3:

1. While the introduction provides an extensive background on honey composition, botanical origins, and regional context, it is overly long. Streamlining this section would help maintain the reader's focus on the study's aims and hypotheses.

Response to Reviewer 3 – Introduction Length

We sincerely thank the reviewer for the valuable feedback regarding the length of the introduction. In response, the section has been streamlined to improve clarity and maintain focus on the study's aims and hypotheses. Specifically:

1. Redundant or evaluative wording has been removed, and overly detailed background information has been condensed.
2. The introduction was revised to adopt a more neutral and factual style in line with PLOS ONE standards.
3. Transitions were simplified to ensure a clearer progression from general background on honey composition and authentication methods to the specific context of Algerian and Polish honeys.
4. The final paragraph now states the study objectives concisely, highlighting the physicochemical and sensory evaluation of Algerian honeys relative to Polish references.
5. The reference list has been updated by incorporating recent citations, including several suggested by the reviewer, and the numbering of references has been adjusted accordingly.

All modifications have been highlighted in green within the revised manuscript for ease of

verification.

2. The Algerian honey samples were collected between 2017–2018, but sensory evaluation occurred in 2025. Although the authors justify stability under refrigerated storage, this unusually long gap warrants more rigorous evidence, such as comparative analyses of fresh versus stored samples, to ensure sensory and physicochemical integrity was indeed preserved.

Response to Reviewer 3 – Temporal Gap Between Analyses

We note that this concern regarding the seven-year interval between physicochemical analyses (2017–2018) and sensory evaluation (2025) was also raised by Reviewer 1. As explained in our earlier response, honey is exceptionally stable under refrigerated storage (4 °C, sealed amber glass containers, protected from light and humidity) (1. Kędzierska-Matysek, M.; Teter, A.; Daszkiewicz, T.; Topyła, B.; Skałecki, P.; Domaradzki, P.; Florek, M. Effect of Temperature of Two-Year Storage of Varietal Honeys on 5-Hydroxymethylfurfural Content, Diastase Number, and CIE Color Coordinates. Agriculture 2025, 15, 652. https://doi.org/10.3390/agriculture15060652, 2. Youssef, M. K. E.; El-Rify, M. N. A.; Ramadan, E. A. and Saleh, A. S. M. The Effects of Heating Treatment and Storage Temperature on Some Physico-chemical Properties of Some Egyptian Honey Types after one Year Storage. J. Saudi Soc. for Food and Nutrition., Vol. 1, No. 2; 2006 (https://japksu.com/index.php/SSFN/issue/download/84/24). No crystallisation, fermentation, or off-flavours were detected at the time of sensory evaluation, supporting the preservation of sample integrity. Importantly, the sensory results therefore represent genuine varietal profiles of Algerian honeys when compared with Polish references, rather than storage-induced artefacts.

We would like to express our sincere gratitude to the Editor-in-Chief and the reviewers for their careful evaluation and constructive suggestions. Their comments have not only improved the clarity and scientific value of the manuscript but have also helped us to better highlight its contribution. We greatly appreciate their efforts and trust that the revised version will now be suitable for publication.

Corresponding authors
Prof. Dr. Hocine Allali
Prof. Dr. Radosław Kowalski

Your sincerely,

Prof. Dr. Radosław Kowalski
